# Apolipoprotein L-1 renal risk variants form active channels at the plasma membrane driving cytotoxicity

**DOI:** 10.7554/eLife.51185

**Published:** 2020-05-19

**Authors:** Joseph A Giovinazzo, Russell P Thomson, Nailya Khalizova, Patrick J Zager, Nirav Malani, Enrique Rodriguez-Boulan, Jayne Raper, Ryan Schreiner

**Affiliations:** 1 Department of Biological Sciences, Hunter College at City University of New YorkNew YorkUnited States; 2 Department of Ophthalmology, Margaret Dyson Vision Research Institute, Weill Cornell MedicineNew YorkUnited States; 3 GenosityIselinUnited States; Weill Cornell MedicineUnited States; DKFZ-ZMBH AllianceGermany

**Keywords:** protein trafficking, membrane channels, ion channel, apolipoprotein l1, polymorphism, Human

## Abstract

Recently evolved alleles of Apolipoprotein L-1 (*APOL1*) provide increased protection against African trypanosome parasites while also significantly increasing the risk of developing kidney disease in humans. APOL1 protects against trypanosome infections by forming ion channels within the parasite, causing lysis. While the correlation to kidney disease is robust, there is little consensus concerning the underlying disease mechanism. We show in human cells that the APOL1 renal risk variants have a population of active channels at the plasma membrane, which results in an influx of both Na^+^ and Ca^2+^. We propose a model wherein APOL1 channel activity is the upstream event causing cell death, and that the activate-state, plasma membrane-localized channel represents the ideal drug target to combat APOL1-mediated kidney disease.

## Introduction

Apolipoprotein L-1 (*APOL1*) is a primate-specific innate immunity gene, (Smith and Malik, 2009) which provides protection against protozoan parasites (Hager et al., 1994; Samanovic et al., 2009) by forming cation channels within the pathogens (Molina-Portela et al., 2005; Thomson and Finkelstein, 2015). APOL1 circulates on specialized high-density lipoprotein particles termed trypanosome lytic factors, which are endocytosed by the parasites (Hager et al., 1994; Rifkin, 1978; Raper et al., 1999). Once inside, APOL1 leads to an ion flux that drives trypanolysis (Molina-Portela et al., 2005; Thomson and Finkelstein, 2015; Rifkin, 1984). Its activity is governed by a two-step process: Activation at acidic pH followed by channel opening at neutral pH (Thomson and Finkelstein, 2015). This mechanism is inhibited in human-infective *Trypanosoma brucei rhodesiense* (Pérez-Morga et al., 2005) and *T.b. gambiense* (Capewell et al., 2013; Uzureau et al., 2013), leading to sleeping sickness.

A molecular arms race between humans and African trypanosomes has led to the evolution of African *APOL1* variants, G1 (rs73885319 - S342G, rs60910145 - I384M) and G2 (rs71785313 - ∆ 388:389 NY) (Genovese et al., 2010), which provide protection against the human infective trypanosomes (Cooper et al., 2017). This resistance, however, significantly increases the risk of developing a spectrum of chronic kidney diseases when two copies of these renal risk variants (RRVs) are present, including focal segmental glomerulosclerosis, hypertension-associated end stage kidney disease, and HIV-associated nephropathy (Genovese et al., 2010; Kopp et al., 2011; Tzur et al., 2010). The RRVs are also associated with sickle cell nephropathy (Ashley-Koch et al., 2011) and lupus nephritis (Freedman et al., 2014), and drive faster progression from chronic kidney disease to renal failure (Parsa et al., 2013). Importantly, 5 million African Americans are estimated to carry two copies of G1 or G2 (Friedman et al., 2011).

The major isoform of *APOL1* encodes a signal peptide (Nichols et al., 2015; Monajemi et al., 2002) and likely traffics along the secretory pathway, thereby allowing for secretion from hepatocytes onto high density lipoprotein particles (Shukha et al., 2017) or localization to the endoplasmic reticulum (ER) and plasma membrane (PM) in other cell types (Cheng et al., 2015; O'Toole et al., 2018; Olabisi et al., 2016; Heneghan et al., 2015). The majority of intracellular APOL1 remains localized within the ER (Cheng et al., 2015). *APOL1* is expressed by several kidney cell types including the podocyte (Nichols et al., 2015; Ma et al., 2015), and multiple studies point to kidney intrinsic APOL1 as the driver of disease (Reeves-Daniel et al., 2011; Lee et al., 2012a), rather than the circulating APOL1 associated with trypanosome lytic factors (Kozlitina et al., 2016). While the discovery of the RRVs provided an explanation for the increased rates of kidney disease in African Americans, there remains little consensus on how the variants cause disease or which pathways to target for therapeutic intervention.

Overexpression of the RRVs in multiple cell lines and transgenic mouse models causes cytotoxicity, however the mechanism responsible remains unclear. It has been proposed that RRV cytotoxicity is mediated by several possible pathways such as autophagy (Wan et al., 2008), lysosomal permeability (Lan et al., 2014), pyroptosis (Beckerman et al., 2017), mitochondrial dysfunction (Ma et al., 2017), impairment of vacuolar acidification (Kruzel-Davila et al., 2017), activation of stress-activated kinases (Olabisi et al., 2016), and ER stress (Wen et al., 2018). This lack of consensus is unsatisfactory and hinders progress towards developing therapeutics. However, whilst these pathways are seemingly unrelated, most are affected by or activated to combat pore-forming toxins (Huffman et al., 2004; Cancino-Rodezno et al., 2009; Kennedy et al., 2009). Therefore, as APOL1 forms cation channels within trypanosomes after endocytosis (Molina-Portela et al., 2005; Thomson and Finkelstein, 2015), we hypothesize cell intrinsic G1 and G2 also form cytotoxic channels, and that this mechanism links the disparate pathways together.

To perform this study, we focused on the channel forming properties of APOL1. Interestingly, APOL1 led to an intracellular accumulation of Ca^2+^ after 72 hr of overexpression in *Xenopus* oocytes (Heneghan et al., 2015), and Ca^2+^ signaling has been associated with the activation of several aforementioned pathways linked to APOL1 (Lee et al., 2012b; Rizzuto et al., 2012; Krebs et al., 2015). Additionally, treatment of African trypanosomes with human serum led uptake of Ca^2+ ^(Rifkin, 1984). The APOL1 channel is permeable to monovalent Na^+^ and K^+^ (Thomson and Finkelstein, 2015), and its trypanolytic activity is inhibited by reducing extracellular Na^+^ (Molina-Portela et al., 2008). As the plasma membrane is already highly permeable to K^+^, we focused on the potential roles of extracellular Na^+^ and Ca^2+^ in driving APOL1 cytotoxicity. We utilized planar lipid bilayers to evaluate APOL1 as a possible non-selective cation channel, and live-cell fluorescent microscopy with the cytoplasmic Ca^2+^ indicator GCaMP6f (Chen et al., 2013) and membrane voltage sensor FliCR (Abdelfattah et al., 2016) to test for increased Ca^2+^ and Na^+^ flux linked to RRV-induced cytotoxicity. Furthermore, utilizing the retention using selective hooks (RUSH) system (Boncompain et al., 2012), in combination with live-cell and immunofluorescence microscopy, we evaluated the importance and timing of events leading up to RRV-induced cell death, including ER exit and the delivery of APOL1 to the PM.

## Results

### Expression of the *APOL1* renal risk variants G1 and G2, but not G0, leads to cell death

As an innate immunity gene, *APOL1* is induced by pro-inflammatory cytokines such as interferons (Nichols et al., 2015). Prolonged courses of interferon treatment caused acute emergence of collapsing focal segmental glomerulosclerosis in a small subset of patients who were revealed to carry two copies of the RRVs upon retrospective genotyping (Markowitz et al., 2010). This has led to the hypothesis that a sustained increase in RRV expression is a cause of APOL1-driven chronic kidney disease (Nichols et al., 2015; Olabisi et al., 2016).

**Figure 1.**
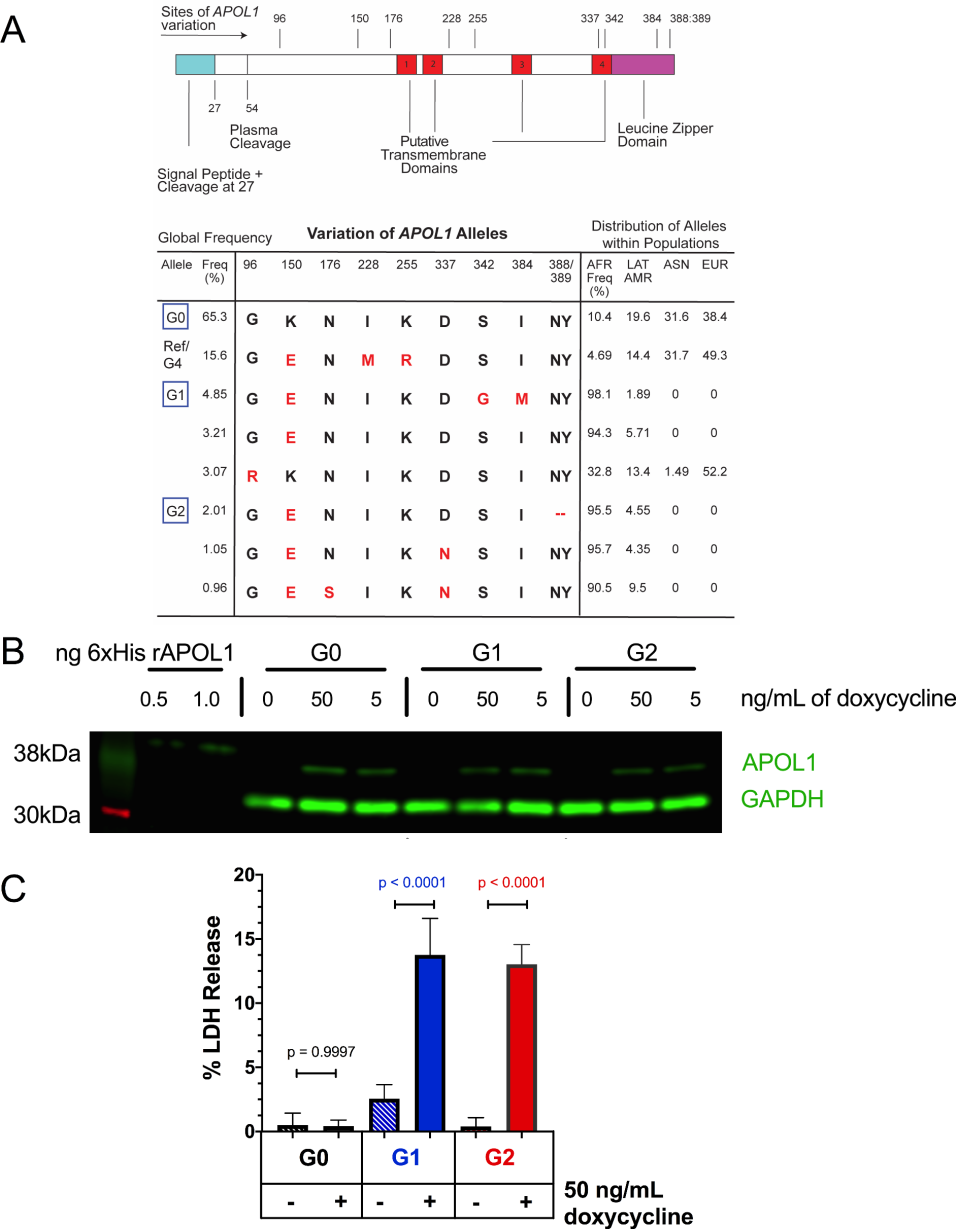
Expression of *APOL1-G1* and *G2* cDNA leads to cytotoxicity in FT293 cells. (**a**) Predicted linear structure of APOL1, using JPred, with major sites of amino acid variation highlighted in red (a deletion is represented as a dash). Haplotypes are organized by frequency in the human population, which is depicted in the left-hand column as Freq (%). The right-hand column represents the distribution of each allele within populations. AFR = African, LAT AMR = Latin America, ASN = Asian, EUR = European. Haplotypes in blue boxes were those used in this study. Data retrieved from 1000 Genomes Project. (**b**) Western blot of whole cell lysates displaying similar levels of protein production between FT293 cell lines. Cells were treated with doxycycline for 4 hr. 6x-His tagged APOL1 was expressed and purified from *E. coli* and used as a positive control. (**c**) Cell death assay displaying the cytotoxicity caused by doxycycline-induced expression of *APOL1-G1* and *G2,* but not *G0*, in FT293 cells. Cells were induced with 50 ng/mL doxycycline for 24 hr, and cytotoxicity was measured via cellular release of lactate dehydrogenase. A two-way ANOVA with multiple comparisons was performed to compare induced and un-induced cells (n = 14).

A Flp-In TREX 293 (FT293) stable cell line was generated to inducibly express *APOL1* variants from a single genetic locus, allowing us to test the effects of sustained *APOL1* expression. These variants are based on the most prevalent haplotypes in the human population (Auton et al., 2015; Figure 1a). Expression of the *APOL1* variants leads to similar levels of protein expression (Figure 1b), and induction of the RRVs, but not G0, leads to cell swelling followed by cytotoxicity after 24 hr of expression (Figure 1c, Figure 1—figure supplement 1a).

**Figure 1—figure supplement 1.**
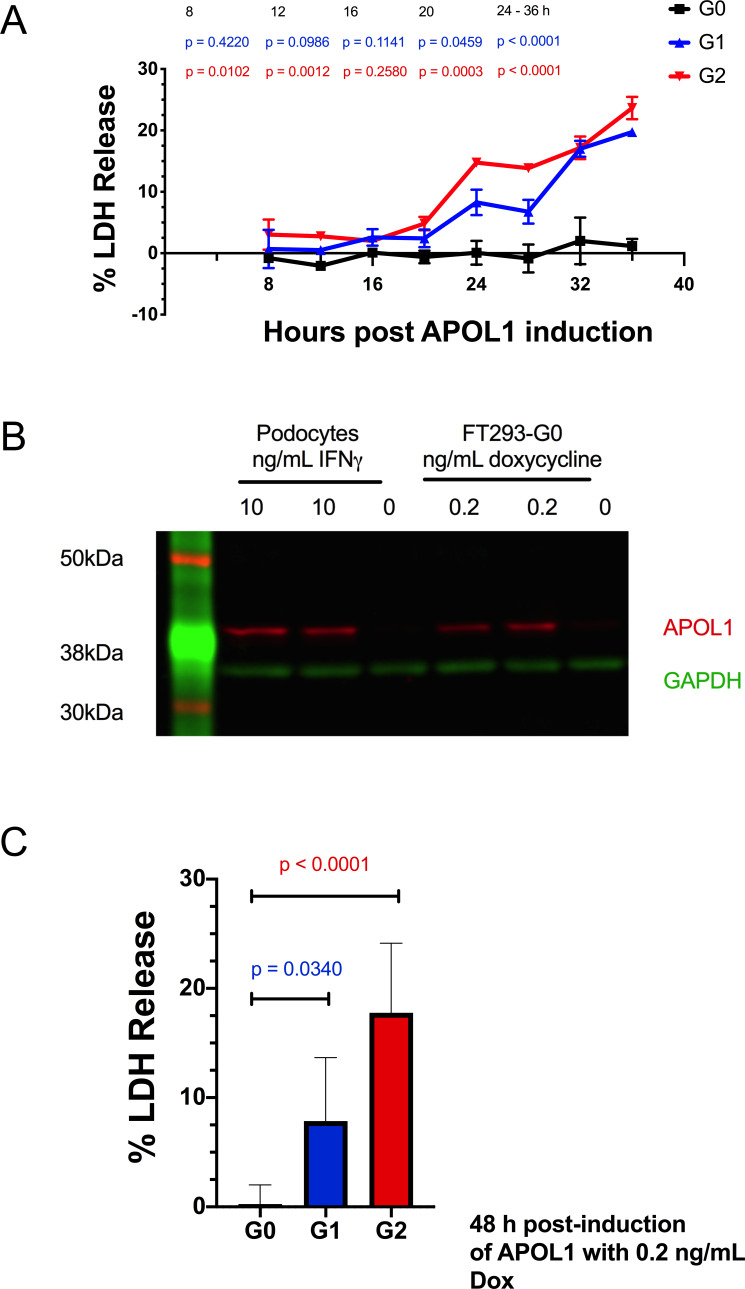
Expressing APOL1 protein at levels found in podocytes leads to RRV cytotoxicity in FT293 cells. (**a**) A time-course of *APOL1* expression in FT293 cells treated with 50 ng/mL doxycycline reveals that RRV cytotoxicity occurs 24 hr post-induction (n = 3). Data are represented as mean ± s.d. (**b**) Western blot of whole cell lysates displaying similar levels of APOL1 protein production between differentiated, conditionally-immortalized human podocytes and FT293-G0 cells. Podocytes were treated with 10 ng/mL interferon- γ and FT293 cells with 0.2 ng/mL doxycycline for 24 hr. (**c**) Expression of APOL1 protein at comparable levels to a podocyte leads to cytotoxicity with G1 and G2. Cells were induced with 0.2 ng/mL doxycycline (n = 7). (**a**) A two-way ANOVA or (**b**) one-way ANOVA were performed with multiple comparisons to compare G0 vs G1 and G2.

It has recently been suggested that overexpression of *APOL1* in cultured cells may not constitute a physiologically relevant model as lower expression levels are not cytotoxic (O'Toole et al., 2018). However, no reference for *APOL1* expression has been established for comparison. To address this point, we titrated APOL1 protein expression in the FT293-G0 stable cell line to obtain similar APOL1 levels found in interferon-stimulated human podocytes (Saleem et al., 2002). The RRVs remained cytotoxic in our model under these conditions, though cell death was delayed due to lower expression (Figure 1—figure supplement 1b–c). These findings indicate that RRV-mediated cytotoxicity in this cell system occurs with levels of protein expression, as quantified by western blot, comparable to that found in interferon-stimulated podocytes, and represents a productive cell culture model to evaluate APOL1-mediated kidney disease.

### APOL1 channels are permeable to Ca^2+^, and the RRVs lead to a cellular Ca^2+^ influx

We hypothesize that the cytotoxicity of APOL1 in mammalian cells parallels its trypanolytic activity, both resulting from its channel-forming properties. Indeed, APOL1 causes cell swelling and dissipation of Na^+^ and K^+^ gradients in trypanosomes (Molina-Portela et al., 2005; Rifkin, 1984) as well as mammalian cells (O'Toole et al., 2018; Olabisi et al., 2016). Furthermore, overexpression of APOL1 in *Xenopus* oocytes led to an intracellular accumulation of Ca^2+^ (Heneghan et al., 2015). As Ca^2+^ is a potent signaling molecule, aberrantly high cytoplasmic Ca^2+^ levels can activate many cell-signaling pathways; eventually its dysregulation leads to cell death.

**Figure 2.**
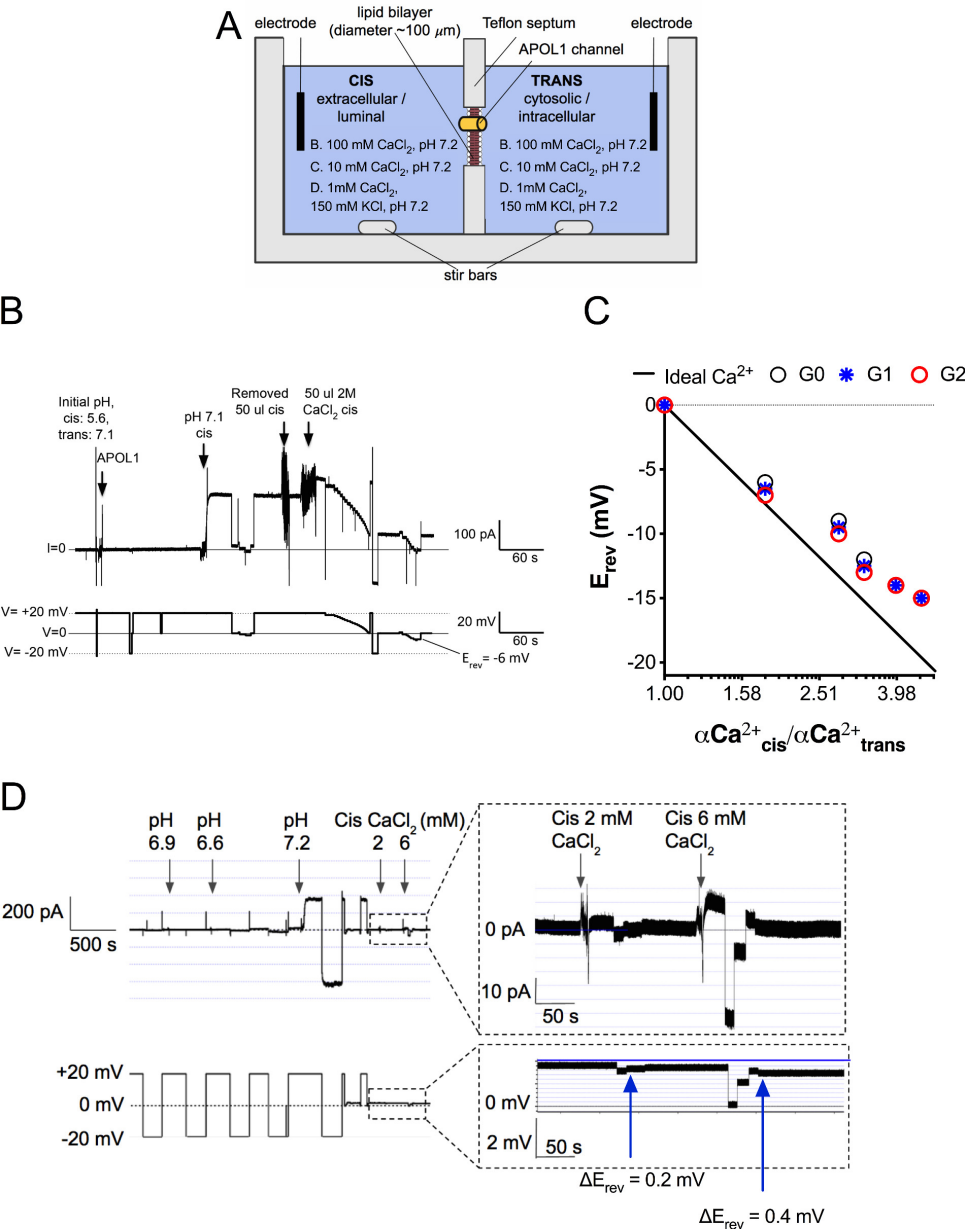
The APOL1 channel is permeable to Ca^2+^. (**a**) Planar lipid bilayer setup. The starting buffer composition for (**b–d**) are shown. During each experiment the composition of the cis side is altered by the experimenter, whereas the trans side is left unaltered. After APOL1 channel formation (typically many thousands per bilayer) a current (pA, upper trace) can be measured in response to a voltage (V, lower trace). In each case the voltage is set by the experimenter. (**b**) Planar lipid bilayer demonstrating that the rAPOL1-G0 channel is selective for Ca^2+^ over Cl^-^. rAPOL1 was added to the cis side at pH 5.6 to drive insertion, which caused a minor increase in conductance that was amplified approximately 450-fold upon cis neutralization (pH 7.1). The voltage required to zero the current (reversal potential, E_rev_) with a 1.95-fold CaCl_2_ gradient was −6 mV, indicating Ca^2+^ selectivity. (**c**) Ca^2+^ versus Cl^-^ permeability did not differ between APOL1 G0, G1 and G2. A conductance was obtained as in **b**, except that the chambers contained symmetrical 10 mM CaCl_2_. The E_rev_ was determined as CaCl_2_ was titrated into the cis side. Plotted are cis/trans Ca^2+^ activity gradients (Robinson and Stokes, 2002) versus E_rev_. Also plotted is the Nernst equation for calcium, which represents ideal selectivity for Ca^2+^ over Cl^-^ (**d**) Ca^2+^ permeability in the presence of excess KCl. Before recording, the cis side was adjusted to pH 6.9 and then 1 µg APOL1 G0 was added to the cis side. APOL1 was allowed to associate with the bilayer for 1 hr and then the cis side was perfused with chamber buffer (150 mM KCl, 1 mM CaCl2, pH 7.2). Once recording began, the cis side was adjusted to pH 6.6, allowing for APOL1 insertion and channel formation. A large increase in the conductance upon re-neutralization of the cis side (pH 7.2) indicates pH-dependent channel opening. E_rev_ (+1.75 mV) was determined by adjusting the voltage until the current read zero. CaCl_2_ was then titrated into the cis compartment to the indicated concentrations. Upon each addition there was an upward shift in the current and the E_rev_ became more negative, indicating Ca^2+^ permeability of the APOL1 channel. The pCa/pK permeability ratio at 2 mM calcium was calculated as 0.6 (See Materials and methods).

The potential Ca^2+^-permeability of channels formed by recombinant APOL1 (rAPOL1) was examined using planar lipid bilayers (Figure 2a). CaCl_2_ was first added to both sides of the bilayer in equimolar amounts. Under conditions where CaCl_2_ was present on both sides of the bilayer, but not KCl or NaCl, the rAPOL1 channel retained its pH-dependent activity, requiring a pH ≤6.0 for irreversible membrane insertion followed by neutralization to open, enhancing conductivity several hundred-fold (Figure 2b; Thomson and Finkelstein, 2015). To examine the ion selectivity of this conductance, we ascertained the reversal potential (E_rev_, the voltage required to zero the current) before and after establishment of a 1.95-fold cis:trans CaCl_2_ gradient. E_rev_ became more negative after CaCl_2_ addition, lowering from −1 mV to −6 mV, indicating selectivity for Ca^2+^ over Cl^-^ and demonstrating that rAPOL1 conducts Ca^2+^ (Figure 2b). All rAPOL1 variants tested are equally permeable to Ca^2+^ (Figure 2c), as well as Na^+^ and K^+^(Thomson and Finkelstein, 2015), indicating that a difference in ion selectivity is not the cause of disease.

We then tested whether APOL1 was measurably permeable to Ca^2+^ at physiological salt concentrations (150 mM K^+^, 1 mM Ca^2+^). Adding an extra 1 mM CaCl_2_ to the cis compartment caused a positive shift in the current and negative shift in the reversal potential (Figure 2d). The pCa/pK permeability ratio at 2 mM Ca^2+^ was calculated as 0.6. This result confirms that APOL1 channels retain Ca^2+^ permeability even in the presence of physiologically relevant KCl concentrations and suggests that APOL1 may lead to a cellular influx of Ca^2+^.

**Figure 3.**
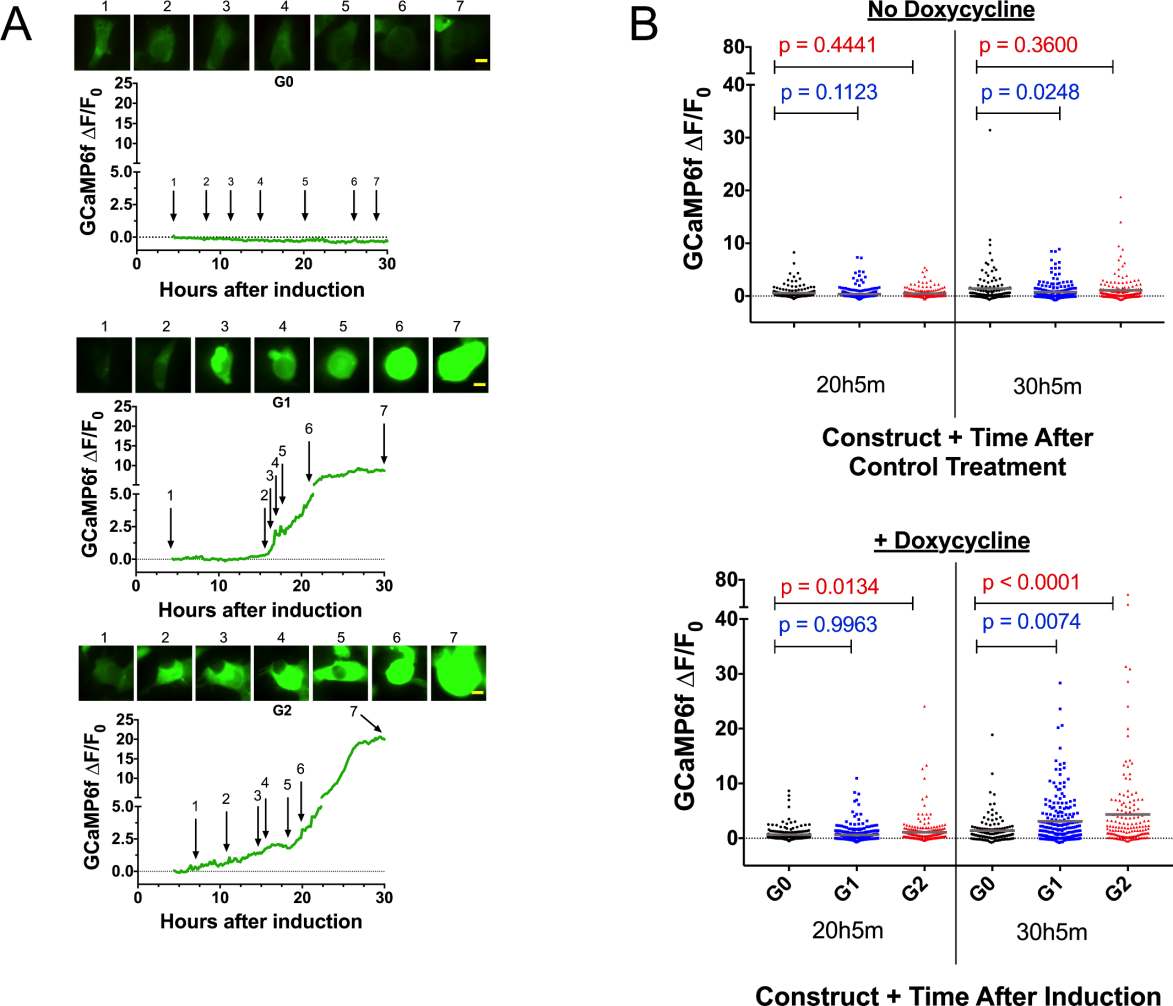
Expression of the RRVs leads to a Ca^2+^ influx that precedes cell swelling and death. (**a**) Fluorescence traces of representative GCaMP6f-positive cells demonstrating that G1 and G2 cause a Ca^2+^ influx prior to cell swelling. GCaMP6f-transfected FT293 cells were incubated with DRAQ7 followed by 50 ng/mL doxycycline to induce *APOL1* expression and then imaged via widefield every 10 min for 4.5–30 hr post induction. Traces represent levels of cytoplasmic Ca^2+^ over time as measured by GCaMP6f fluorescence (no DRAQ7 was observed in depicted cells). Cells are from Video 1. Scale bars = 20 μm. (**b**) High-throughput analysis revealed a significant increase of cytoplasmic Ca^2+^ levels driven by G1 and G2 compared to G0. Each point is the ∆F/F_0_ for an individually tracked cell and bars represent the cell population mean of GCaMP6f fluorescence. Cells were analyzed from 4 fields of view per condition, n = 1748. A one-way ANOVA multiple comparisons test was performed to compare the RRVs with G0 at the indicated timepoints. Figure 3—source data 1.FT283 cells GCaMP6f microscopy, 30 hours after induction one way ANOVA.

To ascertain whether the RRVs lead to a cytoplasmic Ca^2+^ influx upon induction, we transfected the cytoplasmic calcium indicator GCaMP6f (Chen et al., 2013) into FT293 cells. Performing live-cell microscopy with GCaMP6f and cell death marker DRAQ7 allowed us to determine the timing of events between a potential Ca^2+^ influx and changes in cell morphology, plasma membrane integrity, and lysis. Upon induction, cells expressing the RRVs, but not G0, exhibited an increase in Ca^2+^ beginning approximately 12–18 hr after induction (Figure 3a, Video 1). The Ca^2+^ levels increased gradually and occurred several hours prior to cell swelling and membrane blebbing. Cells typically remained swollen for 12–18 hr before lysis (uptake of DRAQ7 was only detected in a few cells within 30 hr of induction, and only after lysis).

**Video 1.** Expression of G1 and G2 leads to a Ca^2+^ influx prior to cell swelling. FT293 cells were transfected with GCaMP6f 24 hr before imaging. Cells were then incubated with 3 µM DRAQ7 and with or without 50 ng/mL doxycycline to induce *APOL1* expression. Cells were imaged via widefield from 4.5 to 30 hr post induction, and dual color images were taken every 10 min. Scale bars = 50 µm.

High-throughput microscopy was performed to analyze individual cells over time and compare the cell populations for changes in Ca^2+^-dependent GCaMP6f fluorescence. There was no difference in mean GCaMP6f fluorescence between the variants without induction. However, after 30 hr of induction we observed a 2 to 3-fold increase in mean GCaMP6f fluorescence with G1 and G2 relative to G0 (Figure 3b and Figure 3—Source data 1). These data confirm that RRV expression leads to an increase of cytoplasmic Ca^2+^ that precedes cell swelling and death, suggesting that Ca^2+^ influx is an early event contributing to cytotoxicity.

### Ca^2+^ influx and cytotoxicity require trafficking of RRVs out of the ER, and the source of Ca^2+^ is extracellular

**Figure 4.**
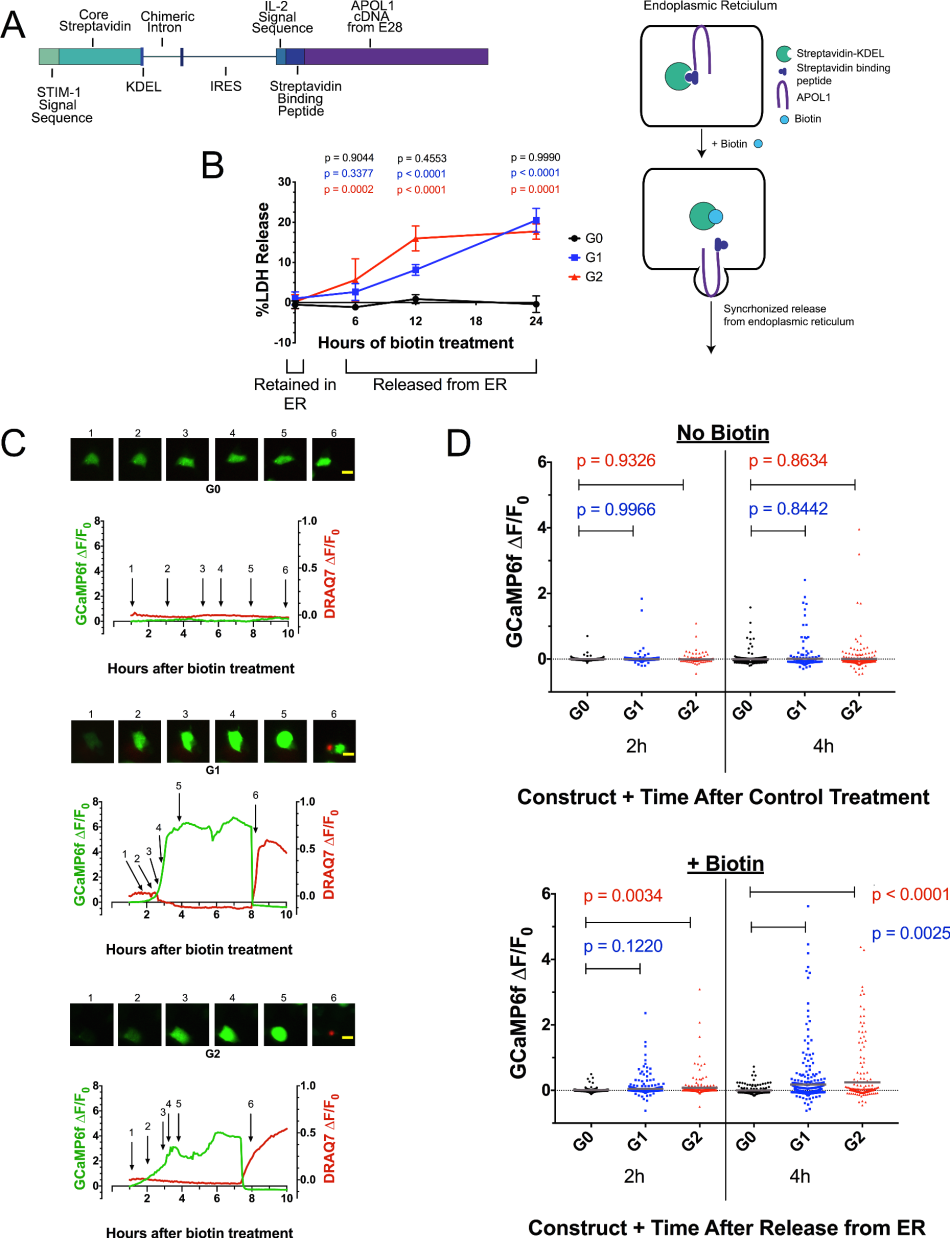
Ca^2+^ influx and cytotoxicity of G1 and G2 requires trafficking from the ER. (**a**) Schematic of the RUSH system. Streptavidin was expressed with a signal peptide and KDEL allowing for localization and retention in the ER lumen along with streptavidin-binding protein (SBP) tagged APOL1. SBP binds to streptavidin causing APOL1 to be retained in the ER until synchronous release is initiated by the addition of biotin. (**b**) Time course showing that RRV cytotoxicity requires trafficking from the ER. 24 hr after transfection, HEK293 cells were treated with or without (0 hr) 80 µM biotin at the indicated times. 48 hr post-transfection, cytotoxicity was measured via release of lactate dehydrogenase. To compare cytotoxicity between biotin treated and untreated (0 hr) for respective genotypes, a two-way ANOVA with multiple comparisons was performed (n = 6). (**c**) Fluorescence traces of GCaMP6f-positive HEK293 cells showing that the G1 and G2-mediated Ca^2+^ influx occurs after trafficking from the ER. GCaMP6f-transfected cells were incubated with DRAQ7 followed by 80 µM biotin to release APOL1 and were then imaged via widefield every 5 min for 1–18 hr post treatment. Cells are from Video 2. Scale bars = 20 μm. (**d**) High-throughput imaging and analysis was performed as in Figure 3b, demonstrating that the G1 and G2-mediated Ca^2+^ influx requires trafficking from the ER. Each point is the ∆F/F_0_ for an individually tracked cell and bars represent the cell population mean of GCaMP6f fluorescence. Cells were analyzed from 3 fields of view per condition, n = 1657. A one-way ANOVA multiple comparisons test was performed to compare the RRVs with G0 at the indicated timepoints.

APOL1 contains a signal peptide (Monajemi et al., 2002) and localizes to the ER (Cheng et al., 2015) and PM (O'Toole et al., 2018; Olabisi et al., 2016), indicating passage through the secretory pathway. To investigate whether RRV-mediated cytotoxicity occurs when localized to the ER or by subsequent trafficking from it, we expressed the *APOL1* variants using the bicistronic RUSH plasmid (Boncompain et al., 2012). RUSH encodes streptavidin targeted to the ER lumen and streptavidin-binding-peptide tagged *APOL1* variants. The streptavidin binds to and retains tagged APOL1 in the ER. Upon treatment with biotin, APOL1 is released from the ER in a synchronous manner (Figure 4a).

**Figure 4—figure supplement 1.**
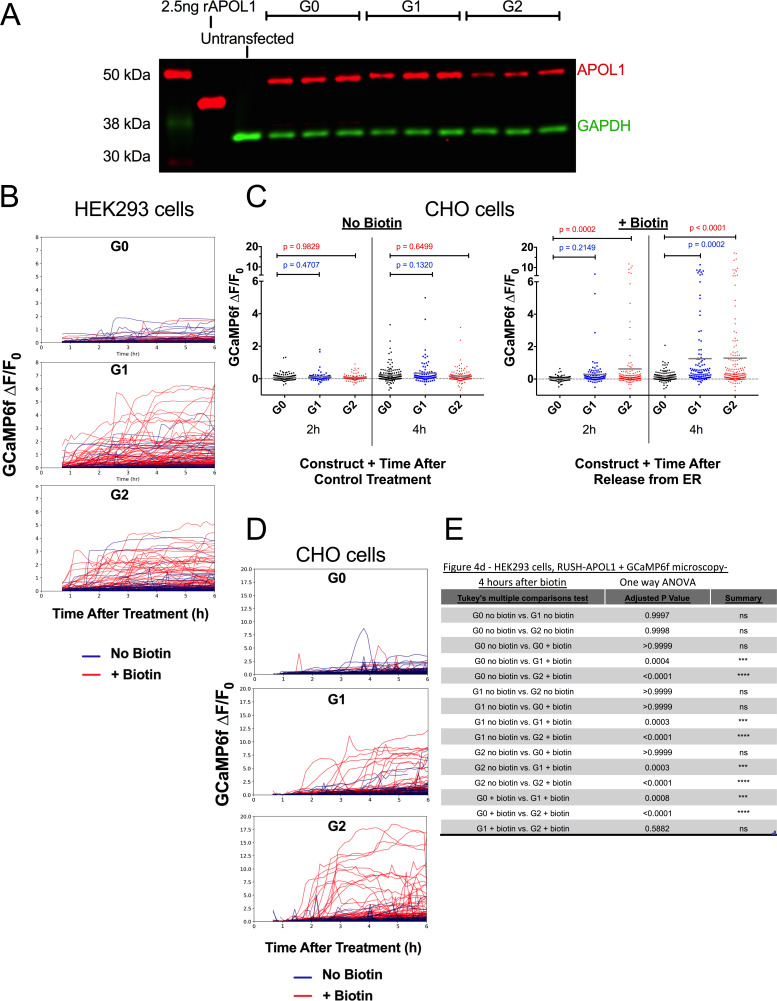
Validation of protein expression and Ca^2+^-driven cytotoxicity of APOL1 in the RUSH system. (**a**) Western blot of whole cell lysates displaying protein expression of RUSH-APOL1 in HEK293 cells 24 hr after transfection. Cells were not treated with biotin. (**b**) Fluorescent traces of all GCaMP6f-positive HEK293 cells in Video 2 and Figure 4d (**c**) High throughput microscopy and analysis was performed as in Figures 3b and 4d, validating in CHO cells the requirement of G1 and G2 trafficking from the ER to mediate a Ca^2+^ influx and cell swelling. RUSH-APOL1 transfected CHO cells were treated with or without 80 µM biotin and imaged via widefield every 5 min for 1–12 hr post treatment. Each point is the ∆F/F_0_ for an individually tracked cell and bars represent the cell population mean of GCaMP6f fluorescence. Representative cells from this analysis can be viewed in Video 3. Cells were analyzed from 3 different fields of view per condition, n = 882. A one-way ANOVA multiple comparisons test was performed to compare G1 and G2 with G0 at the indicated timepoints. (**d**) Fluorescent traces of all GCaMP6f-positive CHO cells from Video 3. (**e**) All cells from 4 hr after +/- biotin treatment in Figure 4d were directly compared via one-way ANOVA.

**Figure 4—figure supplement 2.**
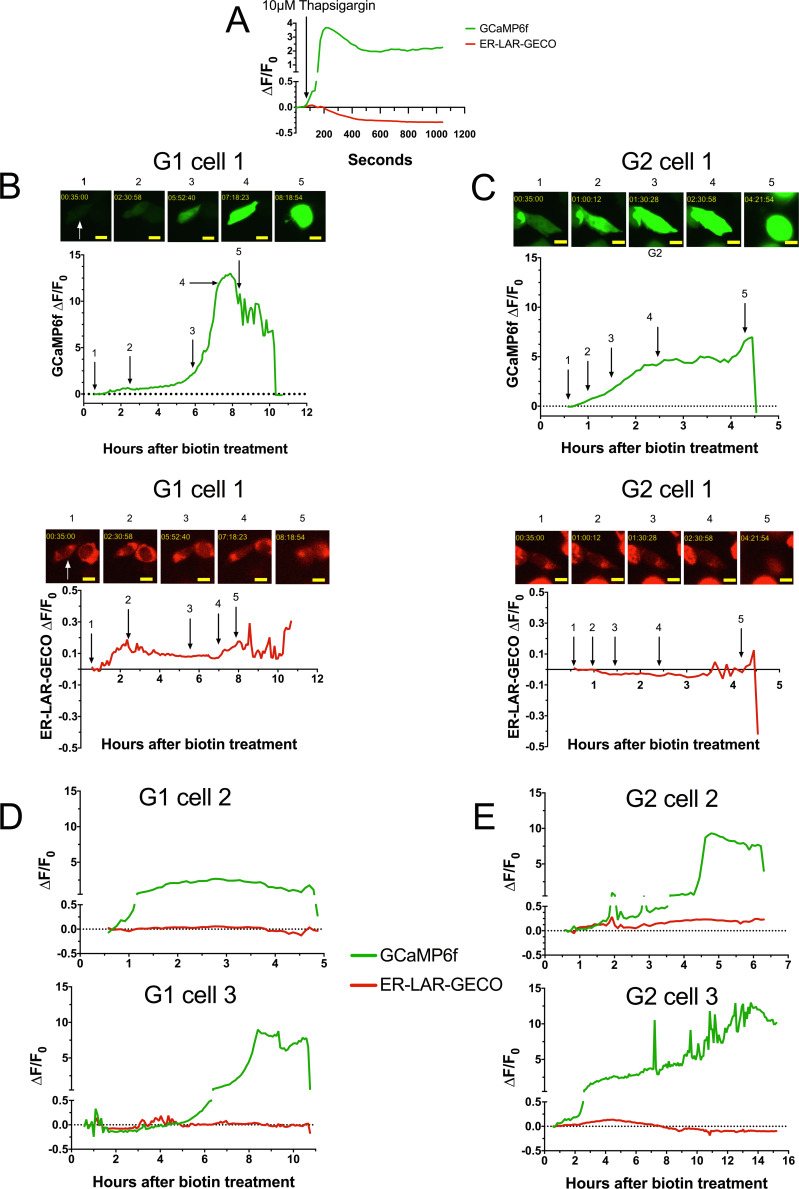
The G1 and G2-mediated cytoplasmic Ca^2+^ influx is not due to ER Ca^2+^ release. (**a**) Validation for the simultaneous use of Ca^2+^ sensors GCaMP6f and ER-LAR-GECO from a representative cell. Co-transfected cells were treated with 10 µM thapsigargin to prevent Ca^2+^ reuptake in the ER, which increases cytoplasmic Ca^2+^ levels (GCaMP6f) while concurrently depleting ER Ca^2+^ (ER-LAR-GECO). Cells were imaged via widefield. (**b–e**) Fluorescence traces revealing that there is no ER Ca^2+^ release during G1 and G2 mediated cytotoxicity. CHO cells were co-transfected with RUSH-APOL1, GCaMP6f, and ER-LAR-GECO, then treated with 80 µM biotin and imaged via widefield every 5 min for 0.5–12 hr post treatment. Cells that displayed the established phenotype of Ca^2+^ influx followed by cell swelling were selected for analysis. Representative cells are from Video 4. A minimum of 5 cells were analyzed per genotype. Scale bars = 10 µm.

RRV cytotoxicity required trafficking from the ER, leading to 20 % cell death 24 hr after biotin-mediated release in transfected HEK293 cells (Figure 4b). In contrast, G0 remained non-toxic after release. No cell death was detected when the APOL1 variants were retained in the ER. Cells producing RUSH-G0 and G1 variants exhibited similar levels of protein production after 24 hr of transfection, though RUSH-G2 expressed approximately 33% less protein (Figure 4—figure supplement 1a), possibly due to its higher cytotoxicity (Figure 4b at 12 hr, 4d at 2 hr with biotin).

We next utilized the RUSH system to determine if the previously observed RRV-mediated Ca^2+^ influx also required exit from the ER by co-transfection with GCaMP6f. Biotin-mediated release of RUSH-G1 and G2 from the ER led to a rapid increase in cytoplasmic Ca^2+^ within 2–4 hr of treatment. Approximately 2 hr after the initial Ca^2+^ influx, membrane blebbing and cell swelling were observed. Cells remained swollen for 4–6 hr until lysis, after which DRAQ7 was detected (Figure 4c, Video 2).

**Video 2.** Expression of RUSH-G1 and G2 leads to Ca^2+^ influx, swelling, and lysis only after release from the ER. HEK293 cells were co-transfected with RUSH-APOL1 and GCaMP6f for 24 hr. Prior to imaging, 3 µM DRAQ7 was added and cells were treated with or without 80 µM biotin to release APOL1 from the ER. Cells were imaged via widefield from 1 to 18 hr post-biotin treatment, and dual color images were taken every 5 min. Scale bars = 20 µm.

High-throughput microscopy was performed revealing a significant increase in the cell population mean of GCaMP6f fluorescence between RUSH-G1 and G2 cells compared to G0. The analysis was limited to 4 hr post-biotin, as nearly all Ca^2+^ influx had begun within that time frame. Without biotin, no difference in GCaMP6f fluorescence was detected. With biotin, RUSH-G1 cells displayed a significant increase in GCaMP6f fluorescence compared to RUSH-G0 at 4 hr, while an increase in RUSH-G2 could be detected as early as 2 hr post-release (Figure 4d, and Figure 4—figure supplement 1b and e). This experiment was reproduced in CHO cells (Figure 4—figure supplement 1c–d, Video 3). These results robustly demonstrate the requirement for G1 and G2 to exit the ER in order to drive a Ca^2+^ influx and cytotoxicity.

**Video 3.** Expression and ER release of RUSH-G1 and G2 leads to Ca^2+^ influx and lysis in CHO cells. CHO cells were co-transfected with RUSH-APOL1 and GCaMP6f for 24 hr. Prior to imaging, cells were treated with or without 80 µM biotin to release APOL1 from the ER. Cells were imaged via widefield from 1 to 12 hr post biotin treatment, and images were taken every 5 min. Scale bars = 20 µm.

The ER is the largest reservoir of intracellular Ca^2+^ (Burdakov et al., 2005), and sequestration and release of ER Ca^2+^ stores plays a pivotal role in many signaling and cell death pathways (Berridge, 2002; Zhivotovsky and Orrenius, 2011). To determine if ER Ca^2+^ release occurs with APOL1 cytotoxicity, cells were co-transfected with RUSH-APOL1, GCaMP6f, and the ER Ca^2+^ sensor ER-LAR-GECO (Wu et al., 2014). The combination of these sensors allows for visualization of ER Ca^2+^ release or lack of re-uptake, as evidenced by treatment with the sarcoendoplasmic reticulum calcium transport ATPase inhibitor thapsigargin (Figure 4—figure supplement 2a). Cells exhibiting the established phenotype of cytoplasmic Ca^2+^ influx followed by swelling were analyzed for fluorescence changes in both sensors. While cytoplasmic Ca^2+^ increases, there is no release of Ca^2+^ from the ER (Figure 4—figure supplement 2b–e, Video 4). The lack of ER Ca^2+^ release indicates that the source of Ca^2+^ in RRV-mediated cytotoxicity is extracellular, possibly conducted via G1 and G2 cation channels at the PM.

**Video 4.** Expression and release of RUSH-G1 and G2 does not induce ER Ca^2+^ release. CHO cells were co-transfected with RUSH-APOL1, GCaMP6f, and ER-LAR-GECO for 24 hr prior to imaging. On the day of the experiment cells were treated with 80 µM biotin and imaged for 0.5–12 hr post treatment. Cells that displayed the established phenotype of Ca^2+^ influx followed by cell swelling were selected. Dual color images were taken every 5 min. Scale bars = 20 µm.

### APOL1 localizes to the PM prior to Ca^2+^ influx

Overexpressed APOL1 has previously been reported to reach the PM (O'Toole et al., 2018; Olabisi et al., 2016; Heneghan et al., 2015). We hypothesize that G1 and G2 must first localize to the PM in order to form cation channels that lead to the observed ion flux and cell swelling. Using confocal immunofluorescence microscopy, we tested whether RUSH-APOL1 would traffic to the PM after biotin treatment, and if localization to the PM occurs within a timeframe before Ca^2+^ influx is first detected in CHO cells (1.75–2 hr post-release, Figure 4—figure supplement 1d, Video 3).

**Figure 5.**
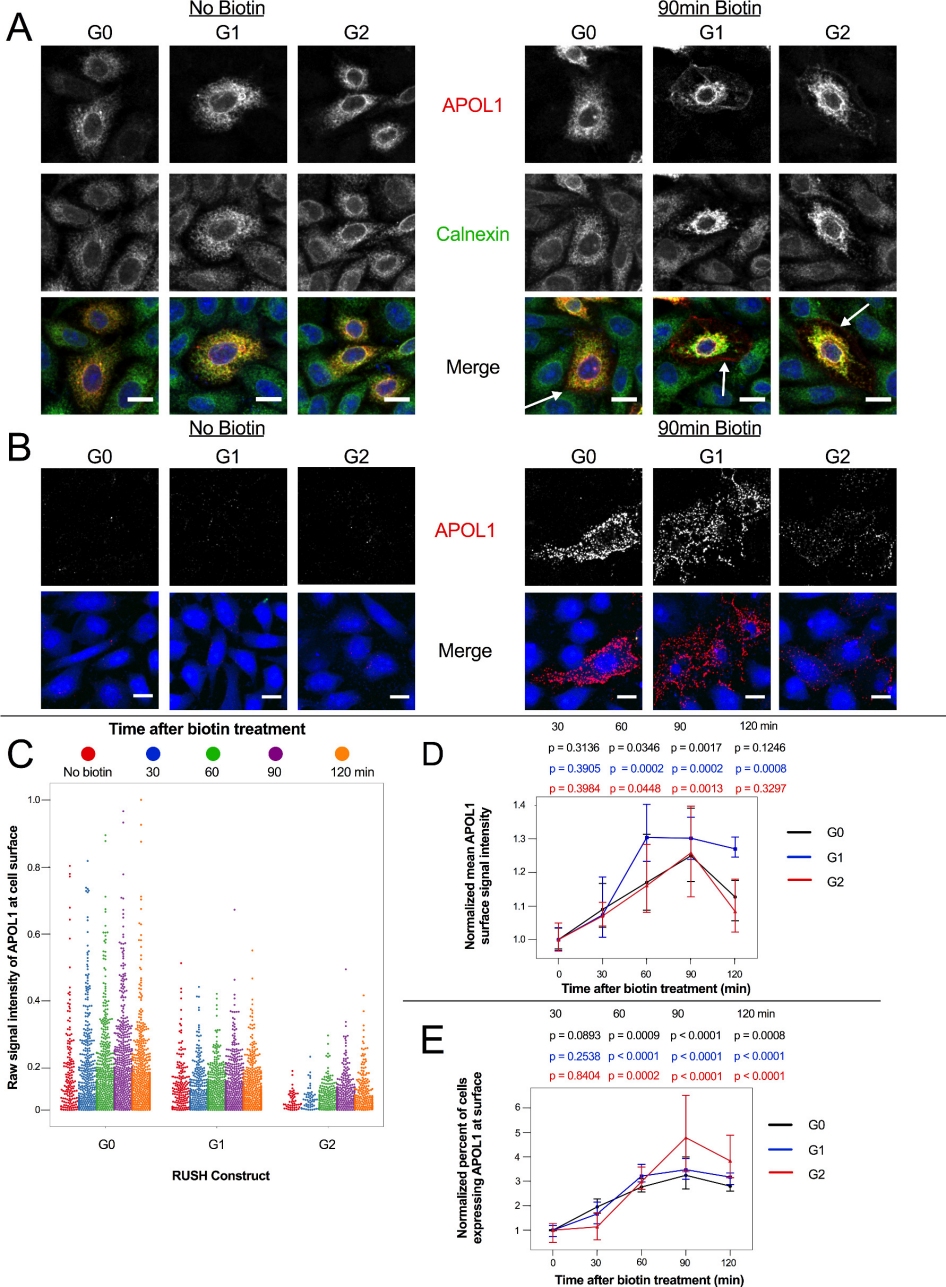
APOL1 traffics to the PM prior to Ca^2+^ influx. (**a**) Confocal images of transfected and permeabilized CHO cells depict RUSH-APOL1 (red) localized to the ER (stained via calnexin, green) without biotin followed by partial PM localization after 90 min of biotin treatment. Representative cells from n = 3 independent experiments. (**b**) RUSH-APOL1 localizes to and forms punctae at the PM within 90 min of biotin treatment. CHO cells were treated and imaged as in (**a**) except without permeabilization. Here anti-calnexin (green) was used as a control for cell permeabilization (depicted in the merged images, no permeabilization was detected). Representative cells from n = 4 independent experiments. (**c**) High-throughput confocal microscopy reveals that RUSH-APOL1 begins localizing to the PM within 60–90 min. Cells were randomly imaged at 20x, capturing ≥10 fields of view per well from 3 replicate wells for each condition. Calnexin signal was used to filter out permeabilized cells. Each dot represents a single cell (n = 462,918 cells analyzed). (**d–e**) RUSH-APOL1 localization to the PM steadily increases until 90 min post release from the ER. (**d**) The mean intensity of all cells in (**c**) was normalized to the respective no biotin controls. (**e**) The percentage of cells expressing RUSH-APOL1 at the PM was determined using a threshold set by untransfected wells, and then normalized to the respective no biotin controls. For analysis of (**d**) and (**e**), a generalized linear model was used to make pairwise comparisons between all samples. Comparisons were performed between biotin treated and untreated cells within each respective genotype. All data are represented as mean ± s.d. (**a–b**) Scale bars = 10 µm.

We found that RUSH-APOL1 traffics to the PM prior to detection of the Ca^2+^ influx, consistent with APOL1 forming cation channels in the plasma membrane. Intracellular antibody staining of ER-retained RUSH-APOL1 cells reveals extensive co-localization with the calnexin-stained ER, as expected (Figure 5a, no biotin). After 90 min of biotin treatment, all three APOL1 variants are detected at the PM (Figure 5a, with biotin, white arrows). Additionally, APOL1 localizes to the peri-nuclear region post ER release, which is suggestive of localization within the Golgi or recycling endosomes after it exits the ER and traffics to the PM (Figure 5—figure supplement 1). Some RUSH-G1 and G2 expressing cells also undergo swelling after 90 min of biotin treatment. In swollen cells, the PM is enriched in APOL1 and the ER is retracted, potentially due to hydrostatic pressure.

**Figure 5—figure supplement 1.**
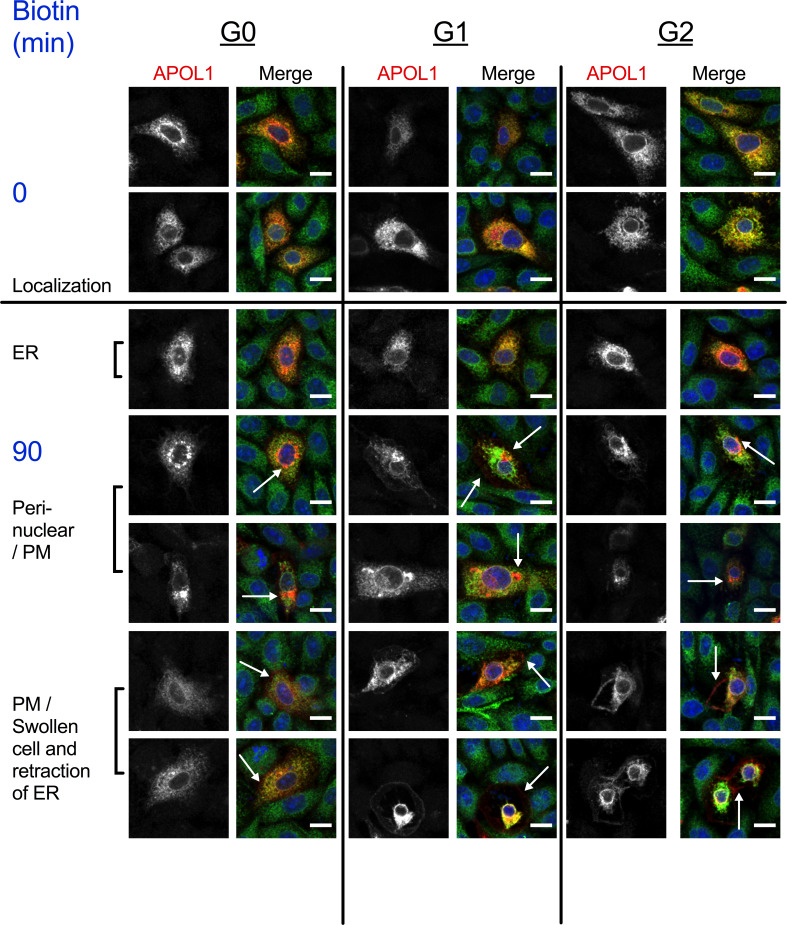
RUSH-APOL1 traffics to the peri-nuclear region and PM post-biotin treatment. Additional confocal images of immunostained CHO cells from Figure 5a. All three APOL1 variants are found in the ER prior to biotin treatment, and then traffic to the peri-nuclear region or PM within 90 min of release (white arrows indicating the plasma membrane and perinuclear compartments). Some G1 and G2 expressing cells start to swell within 90 min, which leads to retraction of the ER. Scale bars = 10 µm.

Correspondingly, via cell surface immunostaining, all RUSH-APOL1 variants were detected at the PM within 90 min of release and displayed a punctate staining pattern (Figure 5b). Due to the leakiness of the RUSH system, APOL1 was also found at the PM of some untreated cells. However, high-throughput microscopy of the transfected cells treated with biotin for 0–120 min revealed a steady increase of APOL1 localization to the PM, peaking at 90 min post-release. After 90 min of biotin treatment, mean APOL1 signal intensity at the PM increased 25–30%, and the number of cells positive for APOL1 staining at the cell surface increased 3–4 fold compared to untreated cells (Figure 5c–e). Less G2 was detected at the surface compared to G0 and G1 (Figure 5b–c), potentially due to the combination of lower protein expression (Figure 4—figure supplement 1a) and higher cytotoxicity (Figure 4b at 12 hr and 4d at 2 hr with biotin). However, cell surface expression still increased in a similar manner compared to G0 and G1. These results demonstrate that RUSH-APOL1 traffics to the PM within the timeframe that a cytoplasmic increase in Ca^2+^ is first detected, and suggests that G1 and G2 form cation channels at the PM as an early event that leads to cytotoxicity.

### RRV-mediated cytotoxicity is driven by the influx of both Na^+^ and Ca^2+^

As a non-selective cation channel, APOL1 may lead to cell death in a variety of ways. It has been postulated that the driver of cell death is APOL1-mediated K^+^ efflux (Olabisi et al., 2016). In that study, Olabisi et al. incubated *APOL1*-expressing 293 cells in ‘CKCM’ media for 24 hr, in which all Na^+^ was replaced by K^+^. The study reported that incubating cells in CKCM reduced RRV cytotoxicity by approximately 50%. Additionally, APOL1 led to K^+^ efflux in trypanosomes (along with a Ca^2+^ influx) (Rifkin, 1984). While APOL1 undoubtedly leads to a K^+^ efflux, the cell is already highly permeable to K^+^ due to the presence of leak channels in the plasma membrane, allowing the cell to rapidly respond to changes in membrane potential or cell volume. Conversely, the cell membrane is minimally permeable to Na^+^ and Ca^2+^, and this permeability could significantly increase in the presence of open G1 and G2 channels at the cell surface. Therefore, we hypothesized cytotoxicity is driven by the influx of Na^+^ and Ca^2+^, rather than solely by the efflux of K^+^.

**Figure 6.**
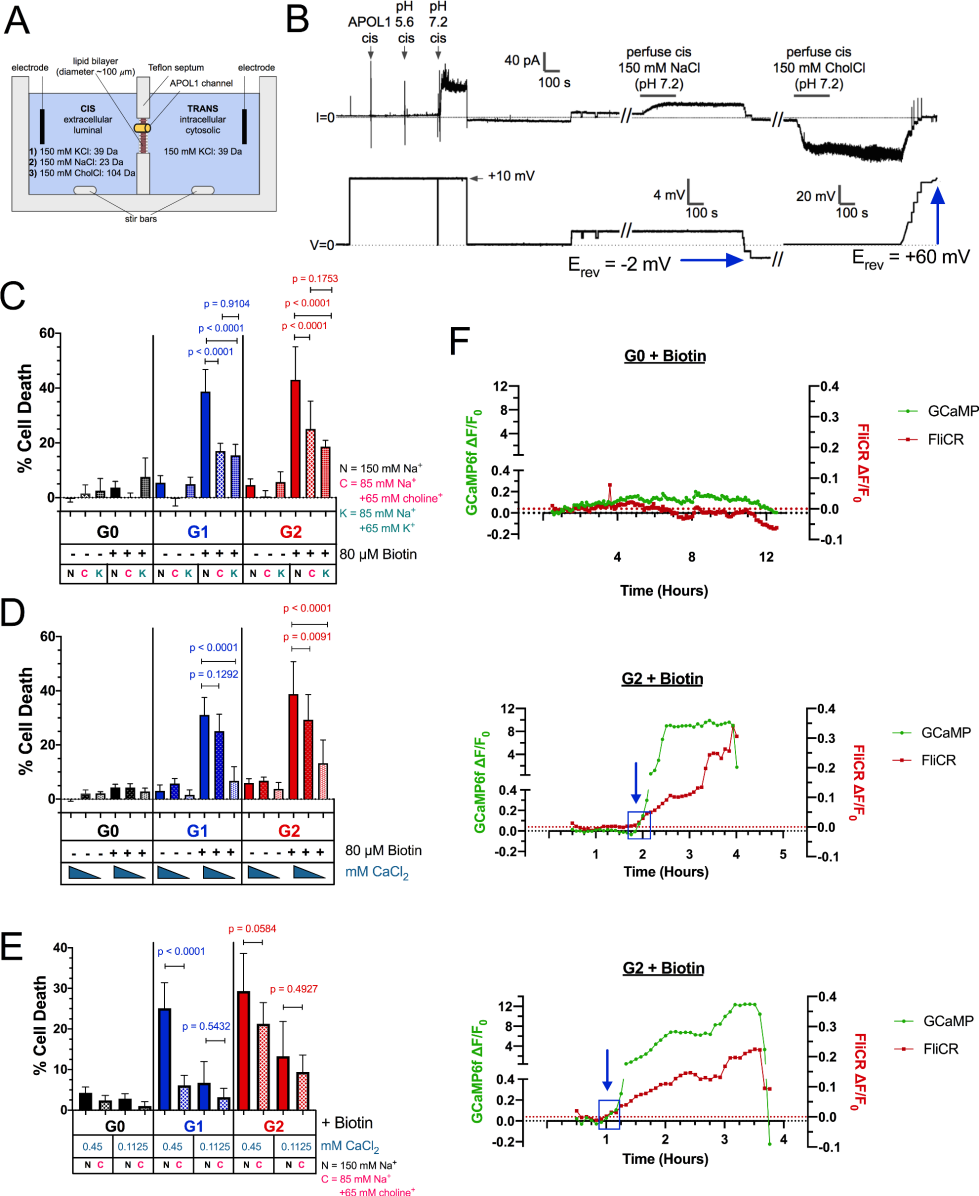
RRV cytotoxicity is driven by the influx of both Na^+^ and Ca^2+^. (**a**) Schematic of the planar lipid bilayer setup showing the sequence of cis buffer perfusions. (**b**) The APOL1 channel is readily permeable to Na^+^, but not choline^+^. In symmetrical KCl solutions E_rev_ was determined as + 1 mV. Then, after cis perfusion with equimolar NaCl buffer (pH 7.2, horizontal bar) there was only a slight change in E_rev_ (E_rev_ = −2 mV; 4 mV scale). In contrast, E_rev_ increased to + 60 mV after exchanging the cis solution for chamber buffer containing equimolar choline chloride. Substituting into the Goldman-Hodgkin-Katz equation (assuming zero permeability to chloride) gives K:Na and K:choline permeability ratios of 1.0:1.1 and 1.0:0.1 respectively. There are two breaks in the record (indicated by //), during which the perfuser was recharged with the appropriate solution. (**c**) The cytotoxicity of the RRVs in RUSH transfected HEK293 cells is significantly reduced by lowering extracellular Na^+^ from 150 mM to 85 mM. The rescue from cytotoxicity was indistinguishable between replacement with either K^+^ or choline^+^ (n = 9). (**d**) RRV cytotoxicity was reduced by lowering extracellular Ca^2+^ from 1.8 mM to 0.45 or 0.1125 mM (n = 12). (**e**) Reduction of both extracellular Ca^2+^ and Na^+^ (replaced by choline^+^) has an additive effect in lowering RRV cytotoxicity, as seen by further rescue from cell death with 0.45 mM Ca^2+^ combined with 85 mM Na^+^ (n = 13). (**c–e**) Cell death was assayed 12 hr post-biotin treatment with the Promega MultiTox fluorescent assay. Two-way ANOVAs with multiple comparisons were performed. (**f**) RRV mediated cytotoxicity is driven by the concurrent influx of both Ca^2+^ and Na^+^. CHO cells were co-transfected with either RUSH-G0 or G2, GCaMP6f, and the membrane voltage sensor FliCR. G2 cells exhibiting the established phenotype of Ca^2+^ influx followed by cell swelling were analyzed for changes in membrane voltage (used as a surrogate for the influx of Na^+^) (n = 21). G0 cells treated with biotin were analyzed for comparison (n = 7). Blue boxes and arrows indicate when the sustained increase in Ca^2+^ initiates. The representative cells from this figure can be viewed in Figure 6—video 1.

We sought to replicate the conditions of the CKCM experiment performed by Olabisi et al. In addition to replacement of Na^+^ with K^+^, we also tested Na^+^ replacement with the larger choline^+^. rAPOL1 channels were tested for permeability of choline^+^ in the planar lipid bilayer system (Figure 6a). Under conditions of symmetrical 150 mM KCl, E_rev_ was +1 mV (Figure 6b), and when the cis-side was perfused and replaced with buffer containing 150 mM NaCl (leaving trans 150 mM KCl unchanged), there was only a slight change in E_rev_ to −2 mV. However, when cis NaCl was perfused and replaced with choline Cl (trans KCl unchanged), there was a significant increase in E_rev_ to +60 mV, indicating conductance of K^+^ from the trans to the cis side, and minimal conductance of choline^+^ (Figure 6b). Substituting into the Goldman-Hodgkin-Katz equation (assuming zero permeability to chloride) gives K:Na and K:choline permeability ratios of 1.0:1.1 and 1.0:0.1 respectively. These results demonstrate that the APOL1 channel is at least 10 times more permeable to K^+^ and Na^+^ than choline^+^.

**Figure 6—figure supplement 1.**
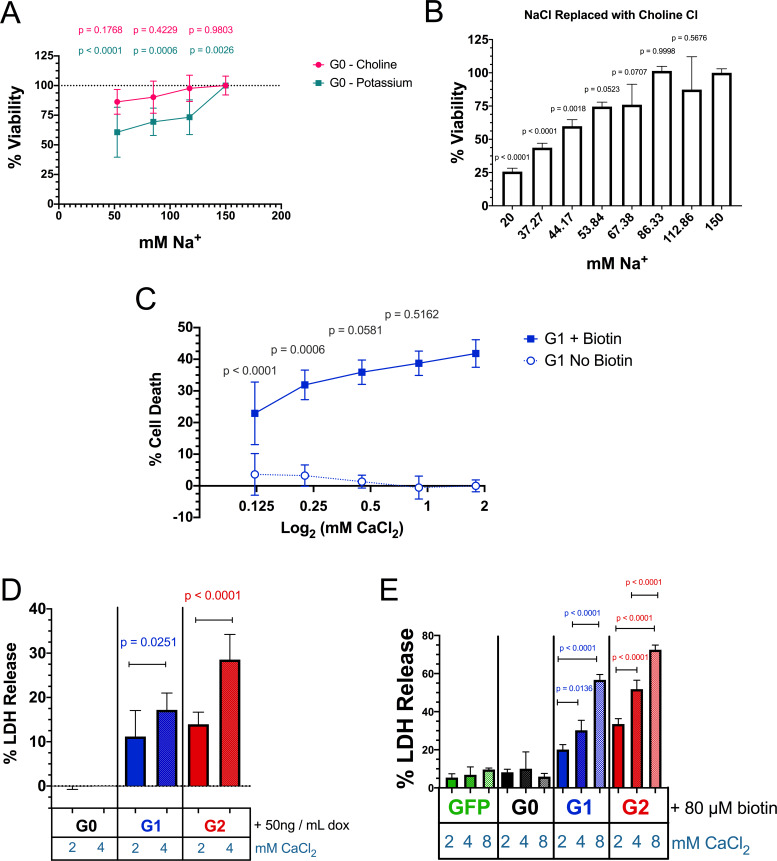
Replacement of Na^+^ with K^+^ significantly reduces cell viability. RRV cytotoxicity is exacerbated with increased extracellular Ca^2+^. (**a**) Replacement of extracellular NaCl with KCl for 12 hr significantly reduces cell viability in RUSH-G0 transfected HEK293 cells (no biotin was added) (n = 6). (**b**) Further replacement of NaCl with choline Cl for 12 hr also reduces cell viability, but only below 86.33 mM Na^+^ (n = 3). (**c**) Reduction of extracellular CaCl_2_ lowers the cytotoxicity of RUSH-G1. (**a–c**) Cell viability and death were measured 12 hr post-biotin treatment using the Promega MultiTox fluorescent assay. (**d–e**) Increasing the levels of CaCl_2_ in the media exacerbates RRV cytotoxicity in both FT293 (**d**), n = 10) and RUSH-transfected HEK 293 cells (**e**), n = 3). Cytotoxicity was assayed 24 hr post induction/biotin treatment via release of lactate dehydrogenase. (**a, c–e**) Analysis was performed with two-way ANOVAs using multiple comparisons. In (**a** and **c**), cells incubated with the indicated salt reductions/replacements were compared with the controls in each treatment group. (**b**) A one-way ANOVA multiple comparisons test was performed.

**Figure 6—figure supplement 2.**
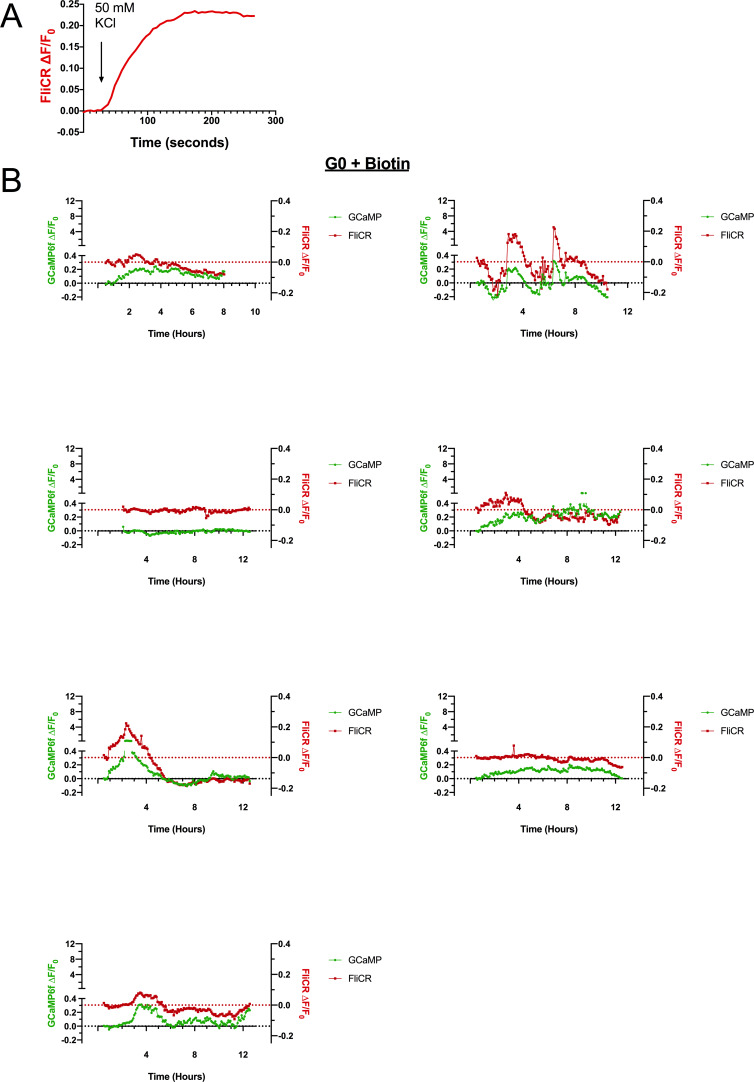
Validation of the FliCR sensor. Release of RUSH-G0 from the ER does not elicit a sustained increase in cytoplasmic Ca^2+^ or membrane depolarization. (**a**) Fluorescent trace of a representative CHO cell transfected with the plasma membrane voltage sensor FliCR. Cells were depolarized by adding 50 mM KCl to the media while imaging. (**b**) RUSH-G0 does not lead to a sustained increase in GCaMP6f or FliCR fluorescence after biotin treatment (n = 7). Some cells elicit temporary increases in cytoplasmic Ca^2+^ and membrane voltage, which may be due to signaling events or passage through the cell cycle.

**Figure 6—figure supplement 3.**
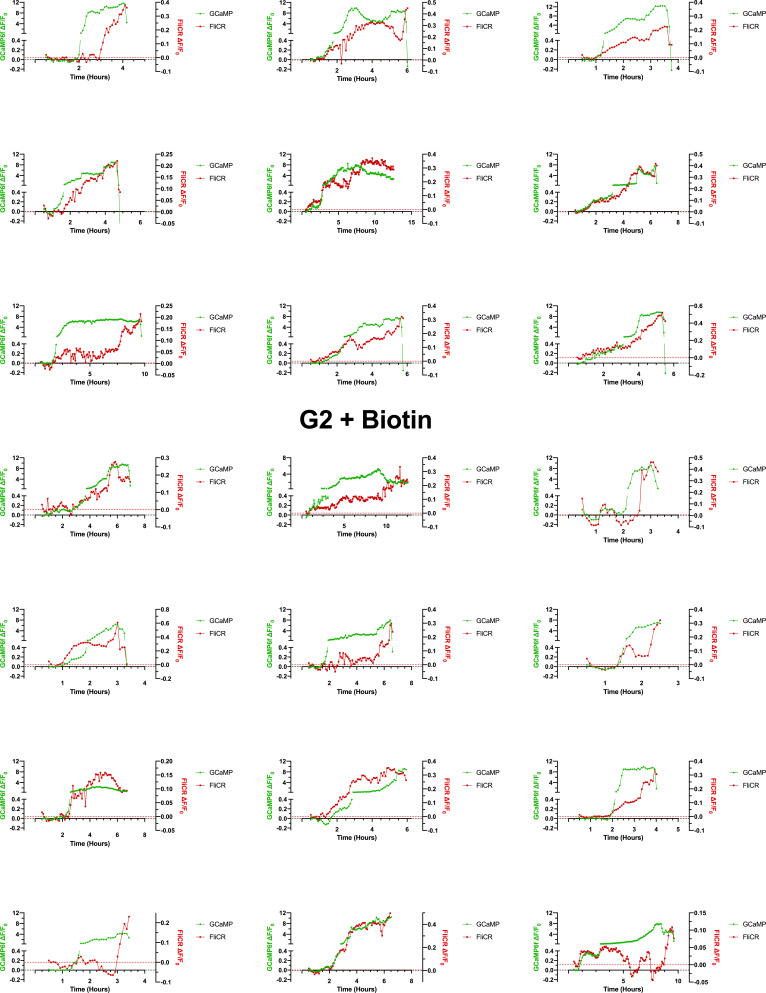
The RUSH-G2-mediated Ca^2+^ influx occurs concurrently with a modest depolarization of the cell (Na^+^ influx), followed by complete depolarization prior to cell death. The G2-mediated Ca^2+^-influx occurs alongside a Na^+^ influx (as measured via membrane voltage) (n = 21). Cytoplasmic Ca^2+^ continues to increase for several hours while the membrane potential either steadily increases alongside it, or becomes erratic and then undergoes depolarization prior to cell death.

**Figure 6—video 1.** The G2-mediated Ca^2+^ influx occurs concurrently with the influx of Na^+^. CHO cells were co-transfected with RUSH-G2, GCaMP6f, and FliCR for 24 hr prior to imaging. On the day of the experiment cells were treated with 80 µM biotin and imaged every 5 min from 0.5 to 12 hr post-biotin (the cells represented here, from Figure 5, underwent lysis by 5 hr). Cells that displayed the established phenotype of Ca^2+^ influx followed by cell swelling were selected. Scale bar = 20 µm.

In order to replace Na^+^ with K^+^ or choline^+^ in the cell culture media, we first tested the effect these conditions have on cell viability. HEK cells were transfected with RUSH-G0 plasmid and incubated for 12 hr in HBSS containing various amounts of NaCl replaced with equal amounts of choline Cl or KCl to make up 130 mM of salt (media contained 150 mM Na^+^). Reducing Na^+^ to 52.5 mM and replacing it with K^+^ led to a 40% reduction in cell viability, whereas replacement with choline^+^ only had a modest effect on cell viability (Figure 6—figure supplement 1a). Further reduction of Na^+^ to 20 mM led to a 75% decrease in viability when replaced by choline+ (Figure 6—figure supplement 1b). Due to these results, we decided to lower Na^+^ to 85 mM for further experimentation, where choline^+^ had no significant effect on viability. A loss of cell viability with K^+^ replacement of Na^+^, however, was unavoidable even at higher concentrations of Na^+^. Additionally, higher amounts of KCl were avoided due to its ability to depolarize the cell.

Reduction of Na^+^ to 85 mM inhibited RRV cytotoxicity by 40–60% (Figure 6c). This level of rescue was similar to the amount reported by Olabisi et al. There was no significant difference between rescue caused by replacement with choline^+^ or K^+^, suggesting that the influx of Na^+^ is a driver of APOL1-mediated cell death, and that it is upstream of the previously reported K^+^ efflux.

As G1 and G2 lead to a cellular influx of Ca^2+^, we tested whether Ca^2+^ itself may act as a driver of cell death. Extracellular Ca^2+^ was serially diluted from 1.8 mM to 0.1125 mM, and a significant reduction in cell death was recorded at Ca^2+^concentrations ≤ 0.45 mM, (Figure 6d and Figure 6—figure supplement 1c). Conversely, increasing extracellular Ca^2+^ exacerbated cell death (Figure 6—figure supplement 1d–e). Reduction of Ca^2+^ and Na^+^ simultaneously (0.45 mM Ca^2+^ and 85 mM Na^+^ supplemented with choline^+^) had an additive effect on inhibiting RRV cytotoxicity (Figure 6e). These results demonstrate that both Na^+^ and Ca^2+^ influx are the initial drivers of RRV-mediated cell death.

Having demonstrated a role for extracellular Na^+^ and Ca^2+^ in RRV-induced cell death, we next sought to measure Na^+^ influx and compare its timing with the influx of Ca^2+^. Due to a lack of any genetically encoded Na^+^ sensors for long-term imaging, we utilized the plasma membrane voltage sensor FliCR as a readout for Na^+^ influx. FliCR expressing cells exhibited an approximately 20–30% increase in ∆F/F_0_ upon depolarization with addition of 50 mM KCl to the extracellular milieu (Figure 6—figure supplement 2a).

CHO cells were co-transfected with RUSH G0 or G2, GCaMP6f, and FliCR and imaged every 5 min for 0.5–12 hr after biotin treatment. No sustained increases in cytoplasmic Ca^2+^ or membrane depolarization were detected in G0-expressing cells (Figure 6f and Figure 6—figure supplement 2b). The initial G2-mediated Ca^2+^ influx occurred concurrently with an increase in membrane depolarization (Figure 6f blue boxes, Figure 6—figure supplement 3, Figure 6—video 1). As Ca^2+^ continued to accumulate within the cytoplasm, cells either exhibited a parallel increase in membrane depolarization, or underwent erratic fluctuations followed by significant depolarization prior to cell death (Figure 6—figure supplement 3).

Na^+^ influx can drive accumulation of intracellular Ca^2+^, either by disrupting the Na^+^/Ca^2+^ exchanger at the PM (Barry et al., 1985) or by depolarizing the cell and causing voltage-gated Ca^2+^ channels to open. However, while these events may contribute to Ca^2+^ accumulation, APOL1 itself can conduct Ca^2+^ (Figure 2a–d) and leads to a large and sustained influx. Indeed, the G2-mediated Ca^2+^ influx was unaffected by Na^+^ replacement with choline^+^ or K^+^ (Video 5). Therefore, the RRVs drive cytotoxicity by directly increasing the membrane permeability of both Na^+^ and Ca^2+^.

**Video 5.** Replacement of NaCl with choline Cl or KCl does not affect the G2-mediated Ca^2+^ influx. CHO cells were co-transfected with RUSH-G2 and GCaMP6f for 24 hr prior to imaging. On the day of the experiment cells were treated with 80 µM biotin and incubated in media containing 150 mM Na^+^ (130 mM NaCl), 85 mM Na^+^ and 65 mM choline^+^, or 85 mM Na^+^ and 65 mM K^+^. The G2-mediated Ca^2+^ influx was unaffected by reduced Na^+^. 3 different fields are shown for each condition. Cells were imaged every 5 min from 0.5 to 12 hr post-biotin. Scale bar = 100 µm.

### The cytotoxicity of all APOL1 variants is dependent upon acid-driven activation

The APOL1 cation channel requires two steps to become functional: an acidic pH to drive irreversible membrane insertion, followed by a neutral pH to open the channel (Figure 2b; Thomson and Finkelstein, 2015). Acidification is also required for trypanolytic activity, as trypanosomes pre-treated with the weak base ammonium chloride are protected against APOL1 (Hager et al., 1994). Within a mammalian cell, APOL1 can encounter acidic and neutral environments by trafficking along the secretory pathway (Paroutis et al., 2004). However, while all three APOL1 variants traffic to the PM and form channels that are permissive to Na^+^, K^+ ^(Thomson and Finkelstein, 2015), and Ca^2+^ (Figure 2c) in a planar lipid bilayer, only G1 and G2 lead to cytotoxicity in our models and cause disease. The existence of a chaperone in mammalian cells that mimics the serum resistance associated protein (SRA) (Limou et al., 2015) found in *Trypanosoma brucei rhodesiense* (Pérez-Morga et al., 2005) has been proposed. SRA directly binds to and inactivates G0, preventing its acid activation, but is evaded by G2 (Thomson and Finkelstein, 2015; Thomson et al., 2014). If G0 is sequestered by an unknown chaperone while trafficking along the secretory pathway, or alternatively if G0 is less sensitive to pH changes relative to the RRVs, this could prevent the insertion event necessary for channel formation.

**Figure 7.**
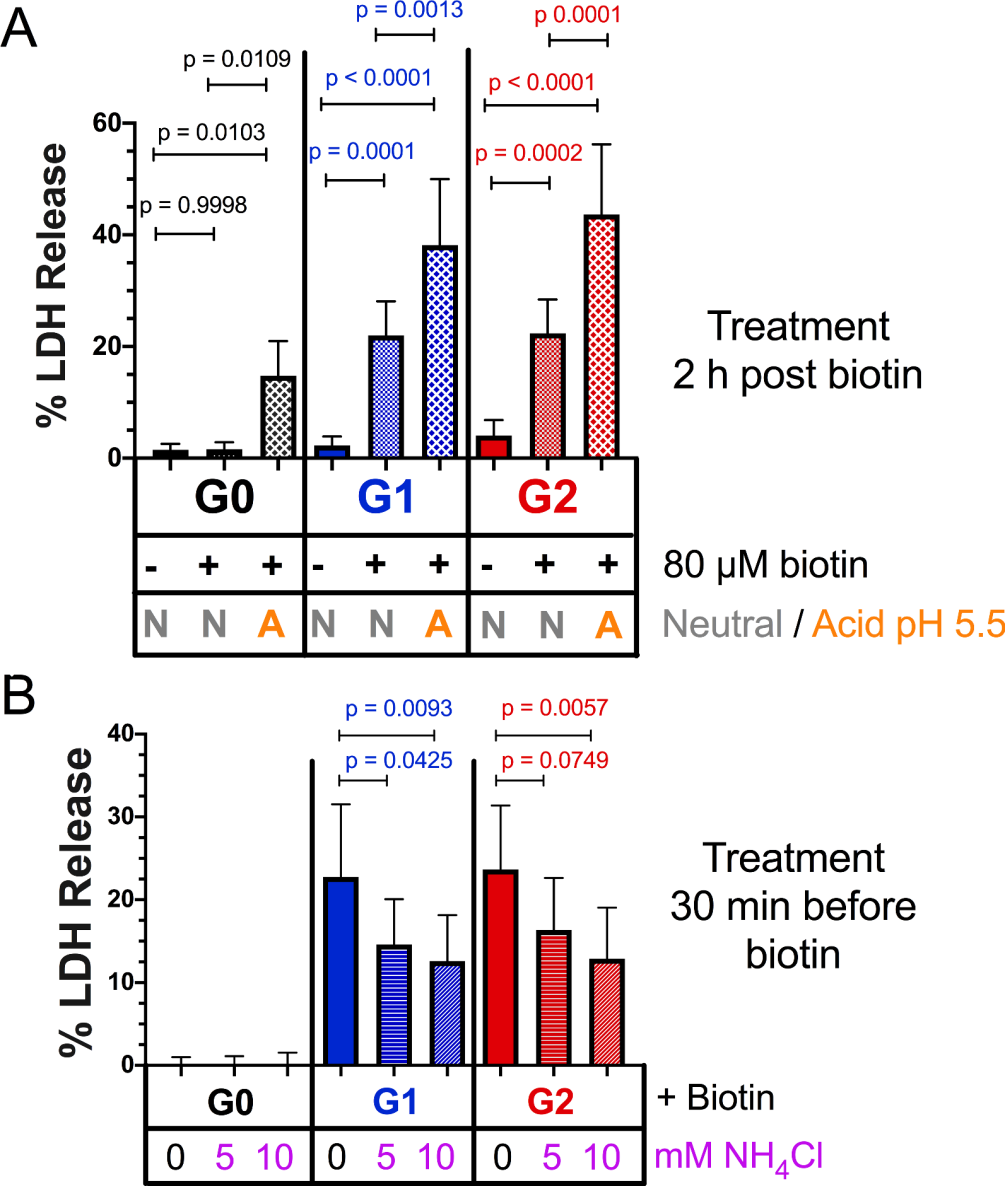
Acidic activation of APOL1 drives channel formation and cytotoxicity. (**a**) Acidification and neutralization of RUSH-APOL1 transfected HEK293 cells causes G0 to become cytotoxic and exacerbates the cytotoxicity of G1 and G2. 24 hr after transfection cells were treated with or without 80 µM biotin. 2 hr post-biotin, cells were incubated with media +/- succinic acid at pH 5.5 for 1 hr followed by neutralization. Cytotoxicity was measured 24 hr post-biotin (n = 13). (**b**) Pre-treatment with ammonium chloride protects against the cytotoxicity of G1 and G2. RUSH-APOL1 transfected HEK293 cells were treated with the indicated amounts of ammonium chloride 30 min prior to biotin treatment. Cytotoxicity was then measured 8 hr after biotin-mediated release (n = 11). (**a–b**) Cytotoxicity was measured via release of lactate dehydrogenase. A two-way ANOVA comparing treated and untreated cells within each respective genotype was performed.

**Figure 8.**
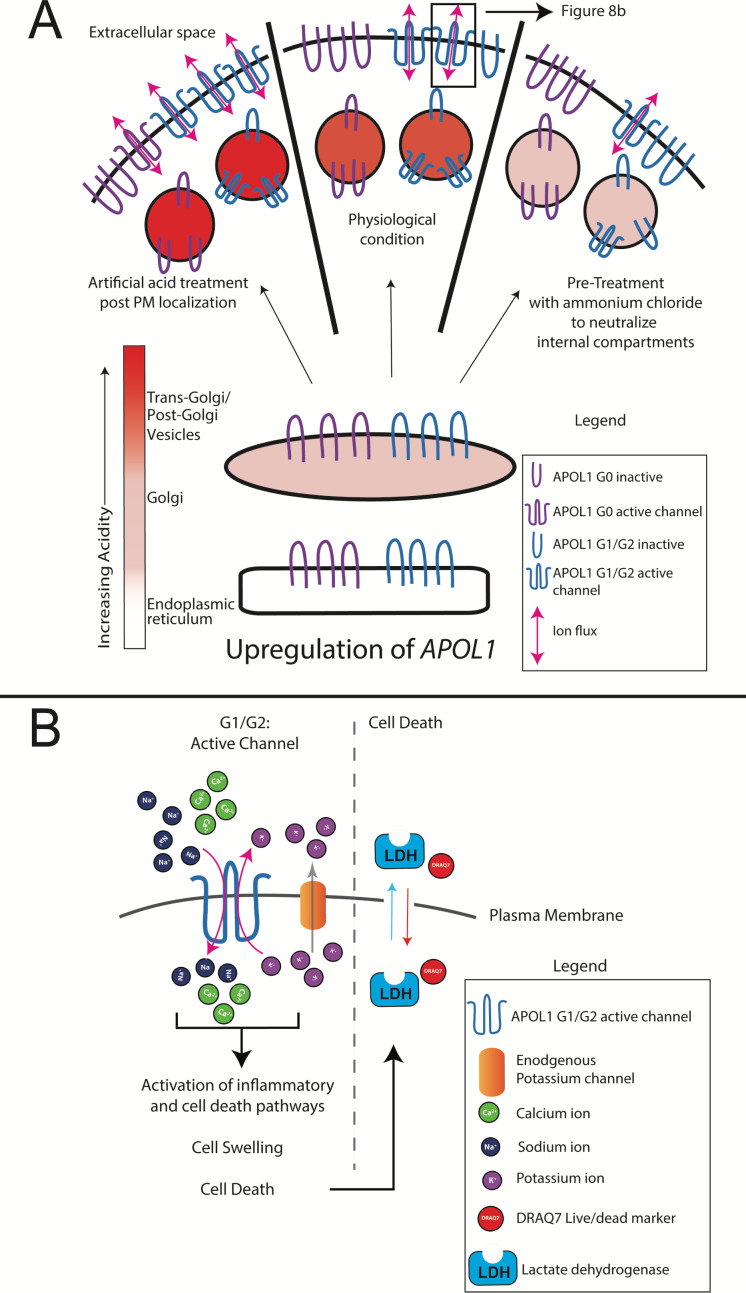
Model of RRV-mediated cytotoxicity: G1 and G2 form cation channels at the PM. (**a**) Proposed model of APOL1 trafficking and cytotoxicity. All variants of APOL1 will traffic to the PM, *en route* they will encounter acidification and neutralization along the secretory pathway, steps required for channel formation. However, while G1 and G2 are able to form cation channels when overexpressed, G0 does not. We hypothesize that G1 and G2 are more sensitive to pH-activation than G0, leading to channel formation. Artificial acidification of cells after localization of APOL1 to the PM caused G0 to become toxic. The increase in G1 and G2 cytotoxicity post-artificial acidification demonstrates that not all APOL1 at the PM is in a channel conformation. Protection against cytotoxicity due to pre-treatment with the weak base ammonium chloride signifies the requirement for acid-activation. (**b**) At the PM, G1 and G2 channels will lead to an influx of extracellular Na^+^ and Ca^2+^, initiating a cascade of events that eventually lead to cell death. Cell death is represented by the assays utilized in this study (release of cytoplasmic lactate dehydrogenase or influx of the live/dead stain DRAQ7).

To circumvent this potential regulatory mechanism along the secretory pathway, RUSH-APOL1 transfected cells were transiently acidified at pH 5.5 and then re-neutralized. This was performed 2 hr post biotin-mediated release, allowing for APOL1 localization to the PM. Under these conditions, RUSH-G0 led to 12.5 % cytotoxicity after release from the ER (Figure 7a). Additionally, the cytotoxicity of RUSH-G1 and G2 increased 1.5 to 2-fold if acidified. Conversely, reducing the acid-activation of APOL1 by pre-treatment with the weak base ammonium chloride significantly lowered the cytotoxicity of G1 and G2 (Figure 7b). The modulation of APOL1-mediated cell death by raising or lowering the pH indicates that not all G1 and G2 at the PM are in an active channel state. These results demonstrate that G0 contains the potential to be innately cytotoxic, however this cytotoxicity is prevented by an unknown mechanism. Conversely, G1 and G2 more readily convert into the active channel state during periods of sustained expression, leading to cell death and disease (Figure 8).

## Discussion

The goal of this study was to investigate the underlying mechanisms driving APOL1-mediated kidney disease. A comprehensive analysis using genetic, biochemical, and microscopy-based approaches revealed that RRV-mediated cytotoxicity first requires trafficking out of the ER to the PM, where they cause a cytotoxic cation flux followed by cell swelling, culminating in lysis. As channel activity leading to a Ca^2+^ and Na^+^ influx is the earliest observed event leading to cell death, and because Ca^2+^ is a potent signaling molecule that can activate many signaling and cell death pathways (Berridge, 2002; Zhivotovsky and Orrenius, 2011), we propose this upstream event links the many APOL1-associated cell death pathways together.

We first replicated previously reported results that the RRVs lead to cytotoxicity (Figure 1c; Olabisi et al., 2016), which was marked by membrane blebbing and a swollen cell phenotype (Videos 1–3), the latter of which was also observed in human serum treated trypanosomes (Rifkin, 1984). Importantly, we used the naturally occurring alleles of *APOL1* (Figure 1a). A study by O’Toole et al. reported that G0 and the RRVs were equally cytotoxic, however their approached utilized artificially synthesized RRVs where C-terminal mutations were introduced into the G4 allele (Figure 1a; O'Toole et al., 2018), which has been shown to have reduced cytotoxicity compared to the naturally occurring haplotype (Lannon et al., 2019). As the lytic activity of APOL1 is sensitive to even single amino acid changes (Cuypers et al., 2016), it is imperative to only use the naturally occurring alleles to draw relevant conclusions regarding kidney disease. While we have used the most prevalent haplotype of G0 with amino acid K150 as our control, it is important to consider the use of G0 E150 for future studies, as G1 and G2 arose in the E150 haplotype background (Figure 1a). It should be noted that there was no difference in cytotoxicity between G0 E150 and G0 K150 as reported by Lannon et al.

We are the first to report that the APOL1 channel is permeable to Ca^2+^, however the selectivity of APOL1 has been controversial. Cl^-^ selectivity was first reported, however this study utilized a truncated rAPOL1 (Pérez-Morga et al., 2005) that was later shown to be non-functional (Molina-Portela et al., 2008). Cl^-^ selectivity was also reported using KCl-loaded large unilamellar vesicles (Bruno et al., 2017), however, Cl^-^ selectivity only occurred at pH 5.0, and they reported APOL1 was K^+^ selective at pH 7.1. While APOL1 may indeed be permeable to Cl^-^, this occurs at pH 5.0 where only a minor current is recorded in planar lipid bilayers. Once neutralized, the current increases several hundred-fold due to the opening of the APOL1 cation channels (Figure 2b; Thomson and Finkelstein, 2015). Opening of these cation channels (but not the minor conductance at acidic pH) was also inhibited by recombinant SRA protein, suggesting a relevance to trypanosome lysis (Thomson and Finkelstein, 2015). Additionally, APOL1 causes the dissipation of Na^+^ (Figure 6f) and K^+^ gradients in animal cells and trypanosomes (Rifkin, 1984; Olabisi et al., 2016; Heneghan et al., 2015), and its trypanolytic activity is inhibited when extracellular Na^+^ is replaced by larger cations that are APOL1-impermeant (Molina-Portela et al., 2005; Figure 6a–b). The results in this paper, together with the previous studies strongly implicate a role for the APOL1 cation channel in biological function, whereas any relevance of Cl^-^ flux remains to be demonstrated.

Through the use of live-cell microscopy with Ca^2+^ sensors GCaMP6f and ER-LAR-GECO and the membrane voltage sensor FliCR, we were able to discern that the upstream event leading to cell death is a cytoplasmic influx of extracellular Na^+^ and Ca^2+^. This was robustly reproduced in multiple cell lines and with the RUSH system, revealing that trafficking of APOL1 out of the ER was required for cytotoxicity. While we described an APOL1-driven Ca^2+^ influx in this study, another group reported no changes in cytoplasmic Ca^2+^(O'Toole et al., 2018). However, their approach utilized the dye Fura-2 measured via fluorescent plate reader at a single timepoint. Using the genetically encoded GCaMP6f and the more sensitive technique of time-lapse, live-cell fluorescent microscopy allowed us to observe the robust Ca^2+^ influx caused by the RRVs.

K^+^ efflux has been proposed as the mechanism that drives APOL1-mediated cell death, however our data demonstrate that this event is likely a response to Na^+^ influx. Unlike K^+^, Na^+^ and Ca^2+^ have a very low permeability across the plasma membrane meaning that APOL1 channels reaching the cell surface will conduct a measurable influx of Na^+^ and Ca^2+^. Na^+^ influx will lead to the observed swelling and depolarization, both of which cause the classic cellular response of K^+^ efflux, which in turn can also affect cell viability via stress activated protein kinases as previously reported (Olabisi et al., 2016). Additionally, the observed protection offered by CKCM media (replacement of all Na^+^ with K^+^) is likely due to the fact that Na^+^ has been removed (Figure 6c). As APOL1 is also permeable to K^+^, it likely contributes directly to the efflux of K^+^, though not to the same degree as Na^+^ and Ca^2+^ influx due to of the presence of other K^+^ channels at the PM. Independent of Na^+^/K^+^ flux, a sustained influx of Ca^2+^ over several hours, which is usually tightly regulated by the cell, can activate a multitude of signaling pathways that may also contribute to cell death.

In order for G1 and G2 to drive a cytotoxic influx of extracellular Ca^2+^, we reasoned that they must reach the PM within the 120 min where an increase of cytoplasmic Ca^2+^ is first detected. Indeed, high-throughput confocal immunofluorescence revealed that all variants of RUSH-APOL1 trafficked with similar kinetics to the PM, which occurred within 60–90 min of biotin treatment (release of ER-retained APOL1). Upon reaching the PM, APOL1 formed a punctate staining pattern. Additionally, localization of APOL1 to the PM was corroborated by other studies (O'Toole et al., 2018; Olabisi et al., 2016; Heneghan et al., 2015). We also report that APOL1 localizes to the peri-nuclear region in some cells, indicating passage through the Golgi and/or endosomes. Interestingly, we observed that the ER had receded from its association with the PM in swollen cells, potentially due to an increase in hydrostatic pressure. A similar phenotype was observed in human submandibular gland cells treated with a hypotonic solution, which caused a loss of ER:PM contact sites (Liu et al., 2010). This may help explain the previously reported case of APOL1-mediated ER stress (Wen et al., 2018).

While all three variants are equally permissive for Ca^2+^ and traffic to the PM in a similar fashion, only the RRVs are cytotoxic. It has been postulated that an SRA-like chaperone may exist to prevent G0 cytotoxicity (Limou et al., 2015), however no such binding partner has been found. We discovered that by artificially acidifying and therefore activating the non-toxic G0 after PM-localization, followed by re-neutralization, G0 led to cell death. Under these conditions RRV-mediated cytotoxicity was also exacerbated. Conversely, pre-treatment with ammonium chloride protected cells from RRV cytotoxicity. This demonstrates the importance of acidic activation for APOL1 channel activity, and that unlike G0, the RRVs more readily arrive at the PM in a channel active state.

It is imperative to elucidate the mechanism that prevents G0 cytotoxicity in order to understand how APOL1-mediated kidney disease manifests. Additionally, future work should determine how an acid-neutral pH gradient across a membrane activates APOL1 to form a channel, which may alter conformation or drive oligomerization. As can be seen in Figure 7a, G0 becomes cytotoxic only after additional acidification and neutralization. It may be that the RRVs are slightly more sensitive to acidification and therefore become activated more readily compared to G0, leading to kidney disease.

In summary, our results demonstrate that the kidney disease associated variants of APOL1 form cytotoxic cation channels at the cell surface. Live-cell analyses demonstrate an initial event leading to cell death is cation flux across the PM, with major ion components being extracellular Na^+^ and Ca^2+^. This ion flux precedes cell swelling by several hours and is therefore the likely driver of cell death. Because many of the reported pathways associated with APOL1 cell death and disease can be activated by pore-forming toxins and/or Ca^2+^ signaling, we propose that the upstream event linking them is APOL1 channel activity at the PM. Taken together, our data strongly suggest that the primary focus for drug development should be prevention of G1 and G2 channel activity and targeting of activated APOL1 channels at the PM within the kidney.

## Materials and methods

**Key resources table:** 

Reagent type (species) or resource	Designation	Source or reference	Identifiers	Additional information
Recombinant DNA reagent (*Homo sapiens*)	APOL1-G0	NCBI	BC143038.1	cDNA
Recombinant DNA reagent (*Homo sapiens*)	APOL1-G1	NCBI	AF305428.1	cDNA
Recombinant DNA reagent (*Homo sapiens*)	APOL1-G2	1000 genomes project, this paper		cDNA *Constructed from mutagenesis from APOL1-G0. Protein coding sequence based off of 1000 genomes data
Recombinant DNA reagent PRG977	PRG977	Regeneron		
Recombinant DNA reagent and transfected construct pcDNA5/FRT/TO	pcDNA5	Thermo Fisher	V652020	APOL1 variants cloned into this plasmid to generate stable cell line (FT293-APOL1_
Recombinant DNA reagent and transfected construct pOG44	p0G44	Thermo Fisher	V600520	
Recombinant DNA reagent and transfected pcDNA6/Tet-repressor	pcDNA6/Tet-repressor	Thermo Fisher	R25001	
Recombinant DNA reagent and transfected Str-KDEL-SBP-EGFP-GPI	RUSH	Addgene	65293	Gift from Franck Perez. APOL1 variants cloned into this plasmid for transfection into cells (RUSH-APOL1). GFP and GPI anchor removed
Recombinant DNA reagent and transfected pGP-CMVB-GCaMP6f	GCaMP6f	Addgene	40755	A gift from Douglas Kim and the GENIE project
Recombinant DNA reagent and transfected CMV-ER-LAR-GECO1	ER-LAR-GECO	Addgene	61244	A gift from Robert Campbell
Recombinant DNA reagent and transfected CMV-FliCR	FliCR	Addgene	74142	A gift from Robert Campbell
Sequence based reagent	APOL1_G0 K150E mutagenesis primers	This paper	PCR primer pair	F:5'TGAAAGAGTTTCCTCGGTTGAAAAGTGAGCTTGAGGATAAC R:5'GTTATCCTCAAGCTCACTTTTCAACCGAGGAAACTCTTTCA
Sequence based reagent	APOL1-G0 E150 Conversion to G1 mutagenesis Round 1 (S243G)	This paper	PCR primer pair	F:5'CGGATGTGGCCCCTGTAGGCTTCTTTCTTGTG R:5'CACAAGAAAGAAGCCTACAGGGGCCACATCCG
Sequence based reagent	APOL1-G0 E150 Conversion to G1 mutagenesis Round 2 (I384M) (Round 1 as template)	This paper	PCR primer pair	F:5'GGAGCTGGAGGAGAAGCTAAACATGCTCAACAATAATTATAAGA R:5'TCTTATAATTATTGTTGAGCATGTTTAGCTTCTCCTCCAGCTCC
Sequence based reagent	APOL1-G0 E150 Conversion to G12 mutagenesis	This paper	PCR primer pair	F: 5'AGCTAAACATTCTCAACAATAAGATTCTGCAGGCGGAC R: 5'GTCCGCCTGCAGAATCTTATTGTTGAGAATGTTTAGCT
Sequence based reagent	Insertion of APOL1 cDNA into pcDNA 5 vector	This paper	PCR primer pair	F: 5'ATGATATCGCCACCATGGAGGGAGCTG R: 5'ATCTCGAGTCATCACAGTTCTTGGTCCGCCTG
Sequence based reagent	Insertion of APOL1 cDNA into RUSH vector	This paper	PCR primer pair	F: 5'ATGCCCTGCAGGAGAGGAAGCTGGAGCGAGG R: 5'ATGCTCTAGACTATCACAGTTCTTGGTCCGCC
Cell line (*Homo sapiens*)	HEK293	ATCC	CRL-1573	
Cell line (*Homo sapiens*)	FlpIn HEK 293	Thermo Fisher		Gift from Dr. Christian Brix Folsted Andersen. Converted into FlpIn TREX293
Cell line (*Homo sapiens*)	Conditionally Immortalized Human podocytes	Saleem et al., 2002		Gift from Dr. Moin Saleem and Dr. Jeffrey Kopp
Cell line (*Cricetulus griseus*)	CHO	ATCC	CCL-61	
Antibody	Mouse anti-APOL1	Proteintech	66124–1-Ig	WB 1:2000 IF 1:800
Antibody	Rabbit anti-APOL1	Proteintech	11486–2-AP	WB 1:5000
Antibody	Rabbit anti-GAPDH	Proteintech	10494–1-AP	WB 1:5000
Antibody	Rabbit anti-Calnexin	Stressgen	SPA-860	IF 1:200
Antibody	Goat anti-mouse 680RD	LICOR	92568070	WB 1:10,000
Antibody	Donkey anti-rabbit 800CW	LICOR	925–32213	WB 1:10,000
Antibody	anti-rabbit Alexa 488 plus	Thermo Fisher	A32731	IF 1:1500
Antibody	anti-mouse Alexa 647	Thermo Fisher	A21236	IF 1:1000
Chemical compound, drug	HCS Nuclear Mask	Thermo Fisher	H10325	IF 1:400
Chemical compound, drug	DRAQ7	Abcam	ab109202	Live cell microscopy 3 µM
Chemical compound, drug	Thapsigargin	Thermo Fisher	T7458	
Chemical compound, drug	Interferon gamma	R and D Systems	285IF100	
Chemical compound, drug	Lactate dehydrogenase assay	Promega	G1781	Cytotox 96 Non-Radioactive Cytotoxicity Assay
Commercial assay kit	MultiTox-Fluor Multiplex Cytotoxicity Assay	Promega	G9201	
Commercial assay kit	Quik Change II Mutagenesis Kit	Agilent	200523	
Software	TrackMate	Tinevez et al., 2017		
Software	Prism	GraphPad		
Software	R-multicomp package	Hothorn et al., 2008		

### Cloning and vector construction

APOL1-G0 (BC143038.1) (Variant containing K150, Figure 1a) linear protein structure was determined using JPred (Cole et al., 2008). APOL1-G0 cDNA in the PRG977 plasmid was subjected to multiple rounds of mutagenesis using the QuikChange II Mutagenesis Kit (Agilent, Santa Clara, CA. 200523), to generate G1 (K150E, S342G, I384M) and G2 (K150E, NY del 388:389). APOL1 cDNA was inserted into the pcDNA5/FRT/TO (Thermo, Waltham, MA. V652020) and the Str-KDEL_SBP-EGFP-GPI (RUSH) (A gift from Franck Perez, Addgene 65293) (Boncompain et al., 2012) mammalian expression vectors. The APOL1 cDNA inserted into the RUSH contained the sequence just downstream of the signal peptide cleavage site at A27.

### Cell culture and transfections

FlpIn 293 cells from Thermo Scientific were first transfected with the pcDNA6/Tet-repressor plasmid and selected with Zeocin (Gibco, Waltham, MA. R25001) at 100 µg/mL and blasticidin (Gibco R21001) at 5 µg/mL. A single clone was expanded to generate all FT293-APOL1 cells. For single copy APOL1 cDNA cell lines, the Flp recombinase vector pOG44 (Thermo V600520) and APOL1 pcDNA5/FRT/TO were co-transfected at a 9:1 ratio. Selection was performed with blasticidin at 5 µg/mL and Hygromycin B (Thermo 10687010) at 150 µg/mL. Foci were pooled and polyclonal cell lines for APOL1 G0, G1, G2, and empty vector were expanded. Cells were then maintained in DMEM (Corning, Corning, NY. 10–017 CM) with 1 mM sodium pyruvate (Sigma, St. Louis, MO. P5280), 10% tet-free FBS, 100 µg/mL Hygromycin B, and 5 µg/mL blasticidin. All experiments were performed in the absence of antibiotics. APOL1 cDNA expression was induced in these cell lines with the addition of doxycycline (Sigma D9891) at 50 ng/mL unless otherwise stated.

HEK293 cells were cultured in DMEM + 10% FBS, CHO cells in F12K (ATCC 30–2004) + 10% FBS, and conditionally immortalized human podocytes (Saleem et al., 2002) in RPMI1640 (Corning 10–040-CV)+ 10% FBS and ITS (Gibco 41400045). Podocytes were maintained at 33C on Type-I collagen-coated plates. For experiments, podocytes were moved to 37C for 5–7 d to allow for differentiation, and then treated for 24 hr with the indicated amounts of Interferon-γ (R and D Systems, Minneapolis, MN. 285IF100). Cells were regularly tested for mycoplasma.

To perform Na^+^ replacement and Ca^2+^ reduction experiments, RUSH-APOL1 transfected HEK293 cells were cultured in Hank's Balanced Salt Solution supplemented with 10% fetal bovine serum, 4 mM L-glutamine, MEM amino acids (Thermo 11130051), MEM non-essential amino acids (Thermo 11140076), and 1 mM sodium pyruvate (approximately 20 mM Na^+^ in total). For Ca^2+^ reduction experiments, HBSS media was supplemented with 130 mM NaCl and CaCl_2_ was serially diluted from 1.8 mM to 0.1125 mM. All plates were coated with 2.5 µg/cm^2^ of fibronectin (Sigma F1141) to promote cell attachment in a low Ca^2+^ environment. For Na^+^ replacement, NaCl in media was reduced from 130 mM (150 mM total Na^+^) to 65 mM and replaced with equal amounts of choline Cl or KCl. Cell death was assayed using the MultiTox-Fluor Multiplex Cytotoxicity Assay (Promega G9201) 12 hr after addition of biotin.

Cells were transfected with Lipofectamine 3000 as per the manufacturer’s instructions. Cytotoxicity was measured via release of lactate dehydrogenase (LDH) using the Cytotox 96 Non-Radioactive Cytotoxicity Assay (Promega, Madison, WI. G1781). To simultaneously measure cytotoxicity and viability, the MultiTox-Fluor Multiplex Cytotoxicity Assay was used in combination with black-walled, optical bottom 96 well plates (Thermo 165305) and read on a Molecular Devices SpectraMax Gemini. Percent cell death was calculated first by determining the cytotoxicity/viability for each sample, followed by using minimum and maximum cell death controls.

### Lysate collection and immunoblotting

Lysates were collected in NP-40 lysis buffer (150 mM NaCl, 1.0 % NP-40, 1 mM EDTA, 50 mM Tris, pH 8.0) with HALT protease inhibitor (Thermo 78430). Total protein content was quantified with the DC protein assay (Bio-Rad, Hercules, CA. 5000112) and samples were diluted into 4x SDS Laemmli buffer with 2.5% β-mercaptoethanol, and equal amounts of protein were loaded into 10% Tris-Glycine SDS PAGE gels (Thermo XP00100). Blocking and antibody incubations were performed in Odyssey PBS Blocking Buffer (LICOR, Lincoln, NE. 927–40000) following the manufacturer’s instructions. Primary antibodies used were mouse anti-APOL1 1:2000 (Proteintech, Rosemont, IL. 66124–1-Ig), rabbit anti-APOL1 1:5000 (Proteintech 11486–2-AP), and rabbit anti-GAPDH 1:5000 (Proteintech 10494–1-AP). Secondary antibodies used were goat anti-mouse 680RD 1:10,000 (LICOR 92568070), and donkey anti-rabbit 800CW 1:10,000 (LICOR 925–32213). Blots were scanned on a LICOR Odyssey Classic.

Electrophysiology rAPOL1 was purified from *E. coli* and analyzed in planar lipid bilayers as previously described (Thomson and Finkelstein, 2015). Briefly, planar lipid bilayers were formed from soybean asolectin, a rich phospholipid mixture from which non-polar lipids had been removed (Kagawa and Racker, 1971). In some experiments, cholesterol was added to increase bilayer stability. The lipid solution in pentane (1.0% asolectin w/v, or 1.5% asolectin, 0.5% cholesterol w/v) was layered on top of the aqueous solutions and the solvent allowed to evaporate. The lipid bilayer was formed by alternately raising the solution volumes above a ~ 100 micron hole in a Teflon septum separating symmetric 1 ml compartments as depicted in Figures 2a and 6a; Qiu et al., 1996. The cis solution was defined as the side to which protein was added. The voltage was reported as that of the cis with respect to the trans. The trans-bilayer current due to APOL1 was measured in response to a voltage which was set by the experimenter. Manipulation of the pH was achieved by adding pre-calibrated volumes of HCl or KOH to the cis buffer.

To test for Ca^2+^ permeability, bilayers were formed between identical solutions of 1) CaCl_2_-buffer: 10 or 100 mM CaCl_2_ (as detailed in Figure 2 legend) 0.5 mM EDTA, 5 mM K-succinate, 5 mM K-HEPES, pH 7.1; or 2) excess KCl buffer: 150 mM KCl, 1 mM CaCl_2_, 0.1 mM EDTA, 5 mM K-MES, 5 mM K-HEPES, pH 7.2. A pH-dependent conductance was obtained with the addition of APOL1 as detailed in Figure 2 and then 1 M CaCl_2_ was titrated into the to the cis compartment at pH 7.2. The reversal potential (E_rev_) was determined before and after CaCl_2_ addition by adjusting the voltage until the current read zero. To calculate permeability ratios of calcium versus potassium (pCa/pK) in the presence of excess KCl we used the following derivation of the Lewis equation as described by Jatzke et al., 2002:

$$\Delta E_{rev}=\frac{RT}{2F}\;In\;(1+\frac{P_{Ca}}{P_{k}}\frac{4[Ca^{2+}]}{\left[K^{+}\right]})$$

where ∆E_rev_ is the change in E_rev_ due to a given change in CaCl_2_ concentration and R, T and F have their usual meanings.

To determine relative permeabilities of monovalent cations K^+^, Na^+^ and choline^+^, a pH-dependent conductance was achieved with APOL1 in excess KCl buffer and then the cis buffer was perfused sequentially with similar solutions in which the KCl was replaced with NaCl and then choline chloride. E_rev_ (the bi-ionic potential) was determined with KCl still present on the trans side. Permeability ratios (pX/pK) were determined by substituting into the Goldman-Hodgkin-Katz equation.

### Live-cell microscopy

All live cell experiments were performed using Fluorobrite DMEM (Gibco A18967-01). FT293 cells were seeded onto black-walled, optical bottom 96 well plates that were freshly coated with 2.5 µg/cm^2^ of fibronectin. Cells were transfected with pGP-CMVB-GCaMP6f (A gift from Douglas Kim and the GENIE project, Addgene 40755) (Chen et al., 2013), and the next day were treated with or without 50 ng/mL doxycycline to induce *APOL1* expression along with the addition of 3 µM DRAQ7 (Abcam, Cambridge, United Kingdom. ab109202). Cells were imaged via widefield every 10 min at 20x.

For microscopy of RUSH-transfected cells, HEK293 and CHO-K1 cells were seeded onto fibronectin coated glass bottom 96-well (Grenier, Kremsmünster, Austria. 655892) or 24-well (Cellvis, Mountain View, CA. P24-1.5H-N) plates. Cells were co-transfected with APOL1 RUSH vectors and one or a combination of the following Ca^2+^ sensors: GCaMP6f, CMV-ER-LAR-GECO1 (A gift from Robert Campbell, Addgene 61244) (Wu et al., 2014), or plasma membrane voltage sensor CMV-FliCR (A gift from Robert Campbell, Addgene 74142) (Abdelfattah et al., 2016). The next day, 80 µM biotin (Sigma B4639) was added to respective wells and cells were then imaged every 5 min at 10 or 20x. Sensor validations for GCaMP6f and ER-LAR-GECO were performed in FT293 cells using the SERCA pump inhibitor thapsigargin (Thermo T7458).

### Immunofluorescence

CHO cells were seeded onto glass bottom, 96 well plates and transfected with RUSH-APOL1 plasmids. 24 hr after transfection, cells were treated with or without 80 µM biotin every 30 min for 0–120 min. For cell surface immunostaining, cells were moved onto ice and blocked with HBSS + Ca^2+^ + Mg^2+^ + 0.5% BSA fraction V, stained with primary antibodies, fixed in 2% formaldehyde (Thermo 28906), quenched with 50 mM NH_4_Cl, and then stained with secondary antibodies. For intracellular staining, cells were permeabilized with 0.075% saponin. Staining was performed with the following antibodies and dyes: mouse anti-APOL1 1:800, rabbit anti-calnexin 1:200 (Stressgen, Farmingdale, NY. SPA-860), anti-rabbit Alexa 488 plus 1:1500 (Thermo A32731), anti-mouse Alexa 647 1:1000 (Thermo A21236), and HCS Nuclear Mask 1:400 (Thermo H10325). Cells were imaged via spinning disk confocal microscopy.

### Microscopy analysis

To measure the Ca^2+^ kinetics in individual cells, fields of view (FOVs) were imaged at random and the corresponding video files were imported into Fiji and analyzed with TrackMate (Tinevez et al., 2017). After automated detection, files were manually curated to remove cells that were dead, overlapping, or those that had migrated out of the FOV. Cells were tracked based upon GCaMP6f signal, and the multi-channel tracking plug-in (https://imagej.net/TrackMate#Extensions) was used to collect data from all other channels within the spot (DRAQ7, ER-LAR-GECO, FliCR). The raw data was then exported and analyzed using R to determine the change in mean fluorescence intensity for each cell using the following equation:

$$(Fluorescence_{Time=X}-Fluorescence_{Time=0})/Fluorescence_{Time=0}=∆F/F0$$

where F_0_ is the average mean fluorescence of the first 3 timepoints for each cell. For analysis with ER-LAR-GECO or FliCR, cells co-expressing GCaMP6f and exhibiting the established phenotype of Ca^2+^ influx and cell swelling were analyzed.

To determine APOL1 trafficking kinetics in non-permeabilized immunostained cells, multiple FOVs were imaged at random at 20x. Image files were exported as maximum intensity Z-projections and analyzed using the scikit-image library in Python (van der Walt et al., 2014). Briefly, NuclearMask stained cell nuclei were segmented and dilated to approximate cell boundaries. Cells were then filtered for cell death through co-localization with the segmented calnexin channel. Finally, APOL1 stain intensity was summed for all cells individually, and the criteria for positive staining of APOL1 was defined as a cell having greater summed APOL1 stained intensity than the maximum summed intensity of any cell within the untransfected image sets. Data from ≥10 FOVs per experimental group were averaged over three replicates and plotted to determine the intensity and percent of cells expressing APOL1 at the PM for each timepoint and genotype.

Preparation of representative images and movies was performed in Fiji. Representative immunofluorescence images are all maximum intensity Z-projections of approximately 10, 0.22 µm slices.

### Microscopes

For live-cell microscopy, a widefield setup was used. Live imaging was performed with a Zeiss (Oberkochen, Germany) Axio Observer with 470, 555, or 625 nm LED excitation along with Zeiss filter cubes 38 (green), 20 (red), and 50 (far-red). Recording was performed using a sCMOS with 6.5 µm pixels (Hamamatsu, Hamamatsu City, Japan. Flash4.0 v2). Live experiments were performed within an incubation chamber at 37C with 5% CO_2_ and humidity. For immunofluorescence, a Zeiss Axio Observer was fitted with a Yokogawa (Musashino, Japan) CSU-X1 spinning disk head. Excitation was performed with a 405, 488, or 639 nm laser. Recording was performed using a back-thinned EMCCD camera with 16 µm pixels (Photometrics, Tucson, AZ. Evolve 512).

### Statistics

All graphing and statistical analyses were performed with Graph Pad Prism or the multicomp package in R (Hothorn et al., 2008). Statistical significance was tested using one or two-way ANOVAs with multiple comparisons tests. All data are represented as mean with 95% confidence interval unless otherwise noted, and all relevant p-values are depicted directly on each graph.

Data derived from quantified immunofluorescence were fit to a general linear model, and a simultaneous multiple comparison procedure between genotypes and timepoints was conducted in R version 3.5.1. P-values were corrected for multiple comparisons via the Benjamini-Hochberg correction (Benjamini and Hochberg, 1995), and significance was set to a minimum of p<0.05.

## Data Availability

All data generated or analysed during this study are included in the manuscript and supporting files. Source data files have been provided for all main figures in Dryad. The following dataset was generated: GiovinazzoJAThomsonRPKhalizovaNZagerPJMalaniNRodriguez-BoulanERaperJSchreinerR2020Data from: Apolipoprotein L-1 renal risk variants form active channels at the plasma membrane driving cytotoxicityDryad Digital Repository10.5061/dryad.1ns1rn8r3PMC729266332427098

## Additional files

Transparent reporting form
