# Supplementary material for: Apolipoprotein L-1 renal risk variants form active channels at the plasma membrane driving cytotoxicity
Source: eLife. 2020 May 19;9:e51185. doi: 10.7554/eLife.51185 (PMC7292663; doi:10.7554/eLife.51185)
Supplement: Transparent reporting form [file elife-51185-transrepform.pdf]

eLIFE
1st Floor
24 Hills Road
Cambridge CB2 1JP, UK
P 01223 855340
W elifesciences.org
T @elife

eLife's transparent reporting form

We encourage authors to provide detailed information *within their submission* to facilitate the interpretation and replication of experiments. Authors can upload supporting documentation to indicate the use of appropriate reporting guidelines for health-related research (see EQUATOR Network), life science research (see the BioSharing Information Resource), or the ARRIVE guidelines for reporting work involving animal research. Where applicable, authors should refer to any relevant reporting standards documents in this form.

If you have any questions, please consult our Journal Policies and/or contact us: editorial@elifesciences.org.

Sample-size estimation

- You should state whether an appropriate sample size was computed when the study was being designed
- You should state the statistical method of sample size computation and any required assumptions
- If no explicit power analysis was used, you should describe how you decided what sample (replicate) size (number) to use

Please outline where this information can be found within the submission (e.g., sections or figure legends), or explain why this information doesn't apply to your submission:

No power analysis was used to determine sample size. A minimum of 3 technical replicates (wells) was always used for each cell-based assay, western blot or microscopy experiment. A minimum of 3 biological replicates was also performed for each of the aforementioned experiments. For all microscopy experiments, the amount of cells analyzed is also listed.

This information is listed within the respective figure legends and the Microscopy Analysis section of the Materials and Methods.

Replicates

- You should report how often each experiment was performed
- You should include a definition of biological versus technical replication
- The data obtained should be provided and sufficient information should be provided to indicate the number of independent biological and/or technical replicates
- If you encountered any outliers, you should describe how these were handled
- Criteria for exclusion/inclusion of data should be clearly stated
- High-throughput sequence data should be uploaded before submission, with a private link for reviewers provided (these are available from both GEO and ArrayExpress)

Please outline where this information can be found within the submission (e.g., sections or figure legends), or explain why this information doesn't apply to your submission:

eLife Sciences Publications, Ltd is a limited liability non-profit non-stock corporation incorporated in the State of Delaware, USA, with company number 5030732, and is registered in the UK with company number FC030576 and branch number BR015634 at the address 1st Floor, 24 Hills Road, Cambridge CB2 1JP | August 2014

The amount of independent experiments (biological replicates) performed is detailed in the respective figure legends for cell-based assays, western blots and immunofluorescence experiments. For live-cell and immunofluorescence microscopy, the amount of individual cells as well as fields of view and replicate wells analyzed is listed in the figure legend with additional information listed in the Microscopy Analysis section of the Material and Methods.

All experiments were independently replicated a minimum of 3 times. For these experiments, 1 representative experiment is shown in the figure. For live-cell microscopy experiments, a minimum of 3 replicate wells for each condition was analyzed within an experiment. Live-cell experiments were replicated across 3 different cell types (FlpIn TREX 293 stable cells, RUSH-transfected HEK cells, RUSH-transfected CHO cells).

All "outliers" were included in analysis. Certain cells were excluded from analysis for live-cell microscopy experiments. This is explained in the Microscopy Analysis section of the Materials and Methods, where certain cells were excluded if " dead, overlapping, or those that had migrated out of the field of view."

Statistical reporting

- Statistical analysis methods should be described and justified
- Raw data should be presented in figures whenever informative to do so (typically when N per group is less than 10)
- For each experiment, you should identify the statistical tests used, exact values of N, definitions of center, methods of multiple test correction, and dispersion and precision measures (e.g., mean, median, SD, SEM, confidence intervals; and, for the major substantive results, a measure of effect size (e.g., Pearson's r, Cohen's d)
- Report exact p-values wherever possible alongside the summary statistics and 95% confidence intervals. These should be reported for all key questions and not only when the p-value is less than 0.05.

Please outline where this information can be found within the submission (e.g., sections or figure legends), or explain why this information doesn't apply to your submission:

Statistical methods are listed within each figure legend, as well as the Statistics section of the Materials and Methods. The results of the statistical analyses are shown within the figures. For each experiment, how the data is represented (e.g. 95% CI and/or mean ± s.d.) and the N values are depicted within the figure legends.

(For large datasets, or papers with a very large number of statistical tests, you may upload a single table file with tests, Ns, etc., with reference to sections in the manuscript.)

Group allocation

- Indicate how samples were allocated into experimental groups (in the case of clinical studies, please specify allocation to treatment method); if randomization was used, please also state if restricted randomization was applied
- Indicate if masking was used during group allocation, data collection and/or data analysis

eLIFE
1st Floor
24 Hills Road
Cambridge CB2 1JP, UK
P 01223 855340
W elifesciences.org
T @elife

Please outline where this information can be found within the submission (e.g., sections or figure legends), or explain why this information doesn't apply to your submission:

Group allocation was not performed for our cell and biochemically based experiments.

However, randomization of sample collection (which sections of the wells were imaged and therefore analyzed) was used for all microscopy experiments via an automated stage. This is depicted in the Microscopy analysis section of the Materials and Methods.

Additional data files ("source data")

- We encourage you to upload relevant additional data files, such as numerical data that are represented as a graph in a figure, or as a summary table
- Where provided, x to a main figure or table
- Include model definition files including the full list of parameters used
- Include code used for data analysis (e.g., R, MatLab)
- Avoid stating that data files are "available upon request"

Please indicate the figures or tables for which source data files have been provided:

The code for the image analysis in figures 2, 3 & 4 can be found:
https://github.com/zagerpatrick/CHO-Cell-Surface-APOL1-IF
The raw images and LDH assay Excel data files for all figures are all curated in Dryad, https://datadryad.org

eLife Sciences Publications, Ltd is a limited liability non-profit non-stock corporation incorporated in the State of Delaware, USA, with company number 5030732, and is registered in the UK with company number FC030576 and branch number BR015634 at the address 1st Floor, 24 Hills Road, Cambridge CB2 1JP | August 2014
